# CDC-like kinase 4 deficiency contributes to pathological cardiac hypertrophy by modulating NEXN phosphorylation

**DOI:** 10.1038/s41467-022-31996-9

**Published:** 2022-07-30

**Authors:** Jian Huang, Luxin Wang, Yunli Shen, Shengqi Zhang, Yaqun Zhou, Jimin Du, Xiue Ma, Yi Liu, Dandan Liang, Dan Shi, Honghui Ma, Li Li, Qi Zhang, Yi-Han Chen

**Affiliations:** 1 Department of Cardiology, Shanghai East Hospital, Tongji University School of Medicine, Shanghai, 200120 China; 2 Key Laboratory of Arrhythmias of the Ministry of Education of China, Tongji University School of Medicine, Shanghai, 200120 China; 3 Institute of Medical Genetics, Tongji University, Shanghai, 200092 China; 4 Research Units of Origin and Regulation of Heart Rhythm, Chinese Academy of Medical Sciences, Shanghai, 200092 China; 5 Department of Pathology and Pathophysiology, Tongji University School of Medicine, Shanghai, 200092 China

**Keywords:** Phosphorylation, Molecular medicine, Cardiac hypertrophy

## Abstract

Kinase-catalyzed phosphorylation plays a crucial role in pathological cardiac hypertrophy. Here, we show that CDC-like kinase 4 (CLK4) is a critical regulator of cardiomyocyte hypertrophy and heart failure. Knockdown of *Clk4* leads to pathological cardiomyocyte hypertrophy, while overexpression of *Clk4* confers resistance to phenylephrine-induced cardiomyocyte hypertrophy. Cardiac-specific *Clk4*-knockout mice manifest pathological myocardial hypertrophy with progressive left ventricular systolic dysfunction and heart dilation. Further investigation identifies nexilin (NEXN) as the direct substrate of CLK4, and overexpression of a phosphorylation-mimic mutant of NEXN is sufficient to reverse the hypertrophic growth of cardiomyocytes induced by *Clk4* knockdown. Importantly, restoring phosphorylation of NEXN ameliorates myocardial hypertrophy in mice with cardiac-specific *Clk4* deletion. We conclude that CLK4 regulates cardiac function through phosphorylation of NEXN, and its deficiency may lead to pathological cardiac hypertrophy. CLK4 is a potential intervention target for the prevention and treatment of heart failure.

## Abstract (web summary)

Phosphorylation catalyzed by kinases is a key event in signaling pathways involved in cardiomyocyte hypertrophy. Here the authors show that the kinase CLK4 ameliorates cardiac hypertrophy by phosphorylating NEXN.

## Introduction

In response to pathological stimuli, such as hypertension and valve defects, the heart undergoes hypertrophic growth characterized by an increase in cardiomyocyte size and re-expression of the fetal gene program^1,2^. While it is initially an adaptive response to maintain cardiac output, prolonged hypertrophic growth is associated with pathological cardiac hypertrophy^1^. Although previous mechanistic studies have identified a multitude of signaling pathways that are crucial to pathological cardiac hypertrophy, there have been no breakthroughs in the treatment of the disease.

CDC-like kinases (CLKs) are an evolutionarily conserved family of dual-specificity CMGC kinases that can autophosphorylate at tyrosine residues and phosphorylate their substrates exclusively on serine/threonine residues^3,4^. The CLK family consists of four members: CLK1, CLK2, CLK3, and CLK4. Functionally, CLKs act as putative high-level regulators of alternative splicing through phosphorylation of serine/arginine-rich domains on splicing factors (SR proteins), thus exerting regulatory effects in a plethora of biological processes, such as oncogenesis, cancer cell migration, and invasion, and virus replication^5–7^. Moreover, CLKs also play roles in processes other than RNA splicing. For instance, in response to feeding and a high-fat diet, CLK2 directly phosphorylates peroxisome proliferator-activated receptor gamma coactivator 1-alpha (PGC-1α) at the SR domain to repress its activity on target genes, through which it controls hepatic gluconeogenesis^8^. Another report has shown that CLKs 1, 2, and 4 are midbody localized, and by directly phosphorylating and activating Aurora B kinase, they play a regulatory role in the Aurora B-dependent abscission checkpoint^9^. Despite the rapid accumulation of knowledge about CLKs, the roles of this family in cardiac physiology and pathology remain to be further explored.

Here, we investigated the functions of CLKs in the heart. Genetic inhibition of *Clk4*, but not *Clk1-3*, stimulated cardiomyocyte hypertrophy. CLK4 was downregulated in failed myocardia, and cardiac-specific *Clk4* deletion (*Clk4*-cKO) in mice caused cardiac hypertrophy and dysfunction. Mechanistically, we identified nexilin (NEXN) as the direct target of CLK4 and the reduction of phosphorylated NEXN mediated the development of pathological myocardial hypertrophy and heart failure in *Clk4*-cKO mice. Together, these results uncover the regulatory role of CLK4 in pathological cardiac hypertrophy and elucidate the underlying mechanisms.

## Results

### CLK4 deficiency was related to pathological cardiomyocyte hypertrophy

**Fig. 1:**
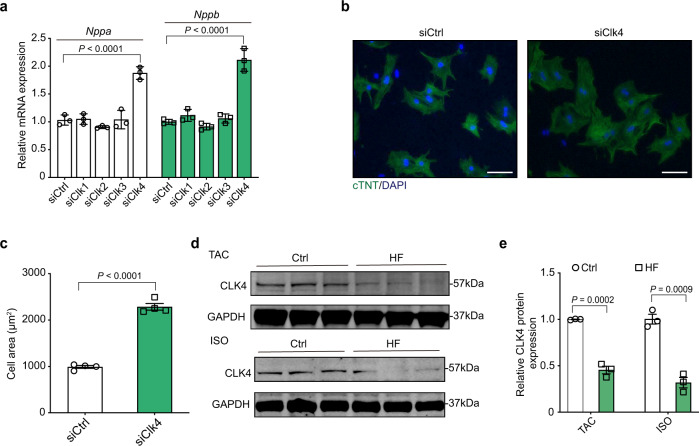
CLK4 regulates cardiomyocyte hypertrophy, and its expression decreases with disease. **a** qPCR detection of the expression of *Nppa* and *Nppb* in NRVMs transfected with control or *Clk1-4* siRNAs. **b**, **c** Representative image of NRVMs treated with control or *Clk4* siRNA for 48 h with cell area quantification. Scale bar: 50 μm. **d** Western blot analysis of CLK4 protein expression in failed mouse hearts subjected to either transverse aortic constriction (upper) or ISO infusion (lower) and in corresponding control mouse hearts. GAPDH served as loading control. **e** Quantification of the western blots in **d**. Ctrl control, NRVMs neonatal rat ventricular myocytes, HF heart failure, TAC transverse aortic constriction, ISO isoproterenol. **a** *n* = 3 biologically independent samples per group; **c**, *n* = 4 biologically independent samples per group; **e**, *n* = 3 animals per group. **a** statistical analysis was performed using one-way ANOVA and Dunnett multiple comparisons test; for **c** and **e**, statistical analyses were performed using upaired, two-tailed Student’s *t* test. Data are presented as the means ± S.E.M.; *P* values are shown in each graph. Source data are provided as a Source Data file.

To investigate the roles of CLKs in cardiomyocytes, we performed a small interfering RNA (siRNA) screen in cultured neonatal rat ventricular myocytes (NRVMs). Quantitative RT-PCR (qPCR) revealed that knockdown of *Clk4* instead of *Clk1-3* induced significant upregulation of natriuretic peptide A (*Nppa*) and natriuretic peptide B (*Nppb*) (Fig. 1a and Supplementary Fig. 2), both of which are well-established markers for pathological cardiomyocyte hypertrophy. Immunofluorescence staining of cTnT further confirmed the hypertrophic growth of cardiomyocytes stimulated by *Clk4* knockdown (Fig. 1b, c). In addition, CLK4 protein levels were significantly reduced in the failed hearts of mice subjected to either transverse aortic constriction or isoproterenol (ISO) infusion (Fig. 1d, e). Importantly, the aforementioned hypertrophic phenotype due to *Clk4* deficiency was also observed in human-induced pluripotent stem cell-derived cardiomyocytes (hiPSC-CMs) (Supplementary Fig. 3). Taken together, these findings indicate that CLK4 deficiency is associated with pathological cardiomyocyte hypertrophy.

### *Clk4* knockout contributed to pathological myocardial hypertrophy and heart failure

**Fig. 2:**
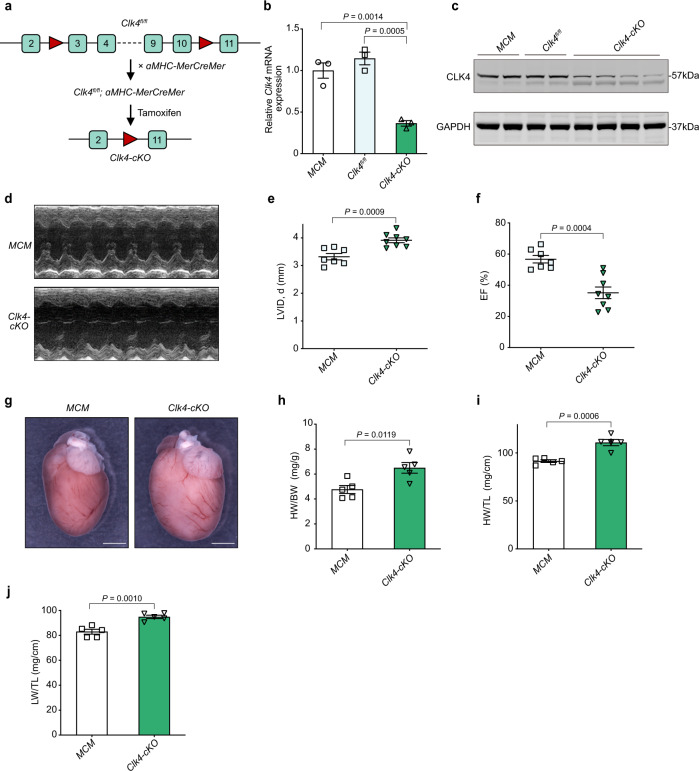
Cardiac-specific *Clk4* knockout (*Clk4*-cKO) leads to cardiac hypertrophy and dysfunction. **a** Schematic diagram of the gene targeting strategy. *Clk4* exons 3–10 are flanked by two loxP sites. *Clk4*^*fl/fl*^ mice were crossed with cardiomyocyte-restricted tamoxifen-inducible transgenic mice (αMHC-MerCreMer, MCM) to generate *Clk4*^*fl/fl*^ × MCM mice. Then, tamoxifen was injected intraperitoneally to induce cardiomyocyte-specific deletion in the adult heart (*Clk4*-cKO). **b** qPCR detection of the expression of *Clk4* mRNA in *Clk4*-cKO and MCM littermate control hearts. Normalized to *Gapdh* expression. *n* = 3 animals. **c** Western blot analysis of CLK4 expression in *Clk4*-cKO hearts. **d** Representative short-axis M-mode images of MCM control and *Clk4*-cKO left ventricles 3 weeks post tamoxifen initiation. **e**, **f** Summary data for the LV internal diameter, diastole (LVID, d) and LV ejection fraction (EF). *n* = 7 animals for MCM and *n* = 8 animals for *Clk4*-cKO. **g** Gross heart images of *Clk4*-cKO and MCM control mice. Scale bar: 2.5 mm. **h** Heart weight-to-body weight ratios (HW/BW). *n* = 5 animals per group. **i** Heart weight-to-tibial length ratios (HW/TL). *n* = 5 animals per group. **j** Lung weight-to-tibial length ratio (LW/TL). *n* = 5 animals per group. For **b**, statistical analysis was performed using one-way ANOVA and Dunnett multiple comparisons test; for **e**, **f**, **h**, **i**, and **j**, statistical analyses were performed using unpaired, two-tailed Student’s *t* test. Data are presented as the means ± S.E.M.; *P* values are shown in each graph. Source data are provided as a Source Data file.

To further investigate the function of CLK4 downregulation in cardiac pathophysiology, we generated a conditional *Clk4* loss-of-function mouse model wherein exons 3–10 of *Clk4* were flanked by loxP sites (*Clk4*^*fl/fl*^). *Clk4*^*fl/fl*^ mice were then crossbred with tamoxifen-inducible Cre-expressing mice under the control of the α-MHC promoter (*α-MHC-MerCreMer*; MCM) to allow cardiomyocyte-specific deletion (Fig. 2a). Mice were born in accordance with Mendel’s law (Supplementary Table 4) and grew normally. Upon Tamoxifen administration to 6-week-old mice, we confirmed an ~70% loss of *Clk4* mRNA and 80% loss of CLK4 protein in *Clk4*^*fl/fl*^ × *MCM* (hereafter referred to as *Clk4*-cKO) mice compared with MCM or *Clk4*^*fl/fl*^ littermates (Fig. 2b, c). Considering the potential impact of Cre expression, we chose MCM littermates as control mice in this study.

**Fig. 3:**
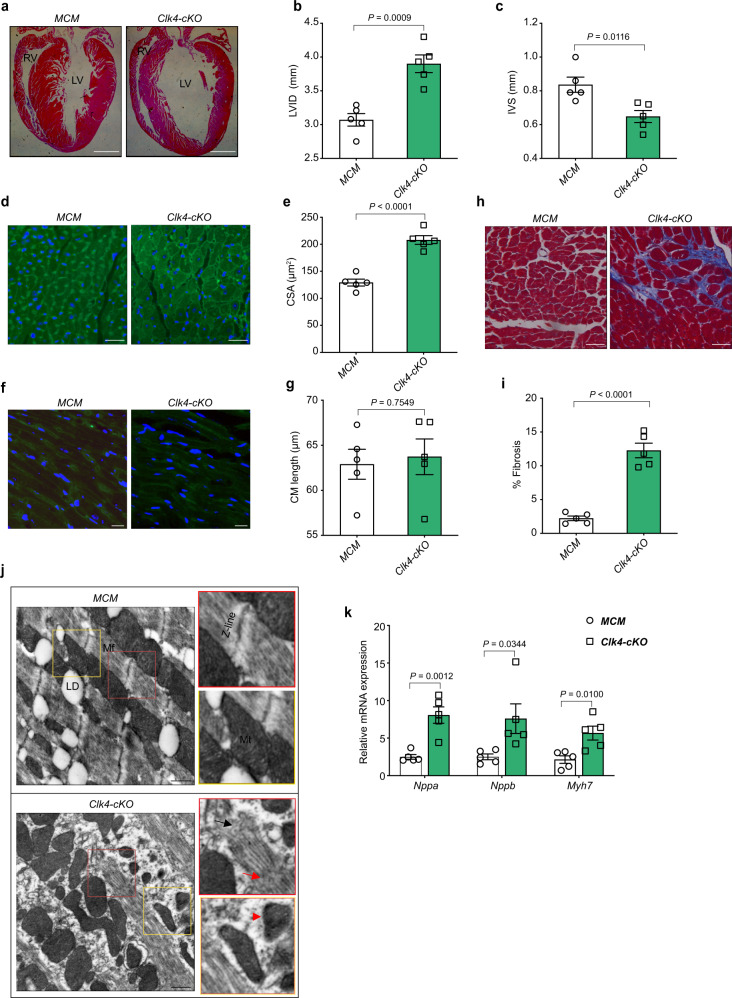
Histological and molecular characterization of *Clk4*-cKO mice. **a** H&E-stained heart sections from MCM control and *Clk4*-cKO hearts. Scale bar: 1 mm. **b**, **c** Summary data for LV internal diameter (LVID) and interventricular septum (IVS) thickness. **d**, **e** WGA staining and quantification of cardiomyocyte cross-sectional area. Scale bar: 50 μm. **f**, **g** WGA staining, and quantification of cardiomyocyte length. Scale bar: 25 μm. **h**, **i** Masson’s trichrome staining of heart sections and quantification of myocardial fibrosis. Scale bar: 50 μm. **j** Transmission electron micrographs comparing control and *Clk4*-cKO hearts. Mt mitochondrion, Mf myofibril, LD lipid droplet, red asterisk, mitochondria; red arrow, blurred and widened Z-disks; black arrow, ruptured myofibril. Scale bars: 500 nm. **k** qPCR analysis of cardiac hypertrophic genes, including *Nppa*, *Nppb* and *Myh7*. For **b**, **c**, **e**, **g**, **i** and **k**, *n* = 5 animals per group. All statistical analyses were performed using unpaired, two-tailed Student’s *t* test. Data are presented as the means ± S.E.M.; *P* values are shown in each graph. Source data are provided as a Source Data file.

Unexpectedly, echocardiography revealed rapidly developed heart failure in *Clk4*-cKO mice, as evidenced by a significant increase in the left ventricular internal diameter, diastole (LVID, d), and a significant decrease in the left ventricular ejection fraction (EF) compared to those of MCM littermate controls (Fig. 2d–f, Supplementary Fig. 4 and Supplementary Table 5). Similarly, heart weight-to-body weight ratio, as well as heart weight to-tibial length ratio, were significantly greater in *Clk4*-cKO mice (Fig. 2g–i), and the lung weight-to-body weight ratio, an indirect indicator of heart failure, were also increased notably in *Clk4*-cKO mice (Fig. 2j). In addition, microstructural examinations, including hematoxylin-eosin (H&E) staining, Masson’s trichrome staining, and wheat germ agglutinin (WGA) staining, all revealed pathological myocardial hypertrophy in *Clk4*-cKO mice, as evidenced by enlarged left ventricles (Fig. 3a–c), increased myocyte size by cardiomyocyte cross-sectional area (CSA), without a significant change in the myocyte length (Fig. 3d–g) and elevated amounts of interstitial fibrosis (Fig. 3h, i). Moreover, ultrastructural evaluation of the *Clk4*-cKO myocardium showed disturbed sarcomere integrity with rupture of myofilaments and blurred Z-disks as well as mitochondrial abnormalities, including fragmentation and disorganization (Fig. 3j). Also, the cardiac hypertrophy in *Clk4*-cKO mice was indicated by dramatic increases in the expression of fetal cardiac genes such as *Nppa*, *Nppb*, and myosin heavy chain 7 (*Myh7*) (Fig. 3k). These data show that cardiac-specific deletion of *Clk4* leads to pathological myocardial hypertrophy and heart failure, demonstrating the direct contribution of CLK4 downregulation in heart pathophysiology.

### CLK4 deficiency altered the myocardial phosphoproteome in mice

**Fig. 4:**
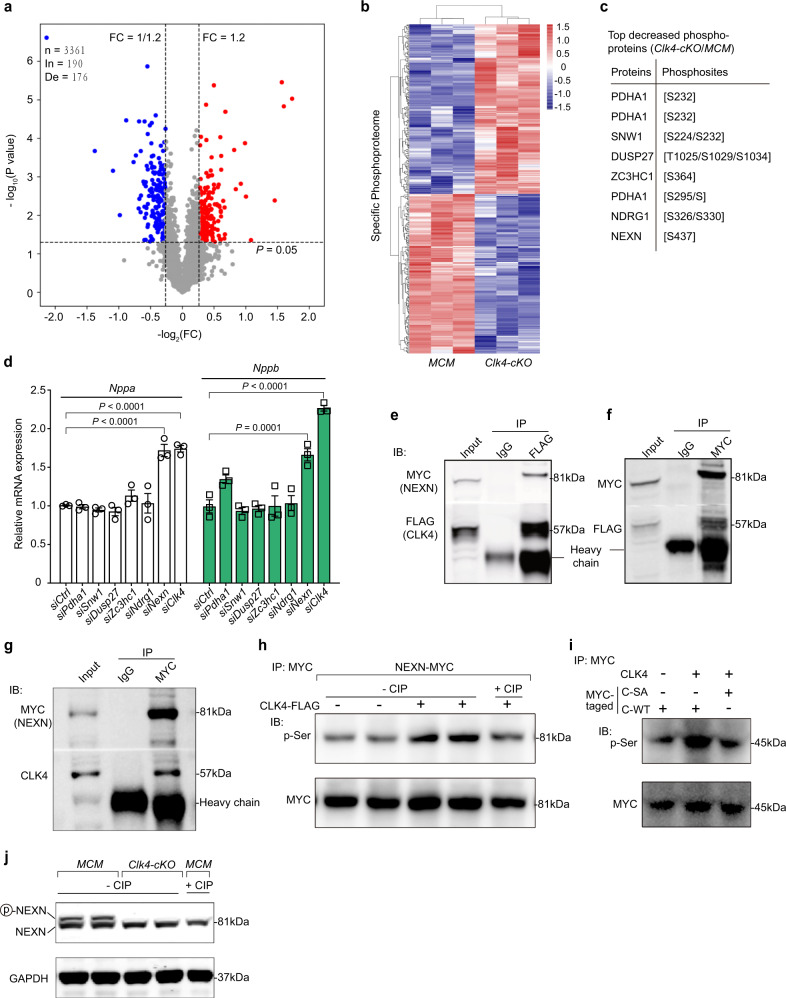
Phosphoproteomic screening of *Clk4*-cKO hearts identifies NEXN as a potential target. **a** Volcano plots showing changes in phosphopeptides in *Clk4*-cKO mice compared with MCM mice, as identified using mass spectrometry (MS). The red dots are peptides with fold-changes (*Clk4*-cKO/MCM ratios) > 1.2 (*P* < 0.05, *t* test), and the blue dots are those with fold-changes < 1/1.2 (*P* < 0.05, *t* test). In, increase; De, decrease. **b** Hierarchical clustering analysis of the specified phosphoproteome. A total of 366 phosphoforms met the aforementioned two criteria. **c** A table showing the top downregulated phosphopeptides and corresponding phosphoproteins obtained from differential gene expression analysis in *Clk4*-cKO hearts. **d** qPCR analysis of cardiac hypertrophic-related genes, including *Nppa* and *Nppb*, in NRVMs transfected with siRNAs targeting *Pdha1*, *Snw1*,*Dusp27*, *Zc3hc1*, *Ndrg1,* and *Nexn*; here, *Clk4* siRNA was used as a positive control. **e**–**g** Coimmunoprecipitation of CLK4 with NEXN in NRVMs transfected with CLK4-flag and NEXN-myc (**e**, **f**), and in vivo myocardium infected with AAV NEXN-WT (**g**). The input represents 6% of the whole-cell lysate used for each immunoprecipitation. IgG was used as a negative control. **h** Representative western blots showing that CLK4 increased the serine phosphorylation level of NEXN in NRVMs. Samples incubated with calf intestinal phosphatase (CIP) served as a negative control. **i** Typical western blots illustrating that CLK4 phosphorylated the wild-type NEXN fragment (amino acids 295–671) but not the mutant type (S437A) in a cell-free system. **j** Western blot detection of the phosphorylated NEXN in *Clk4*-cKO mouse myocardia using a Phos-tagged acrylamide gel. For **a**, **b**, *n* = 3 animals per group; for **d**, *n* = 3 biologically independent samples per group. For **a**, statistical analysis was performed using unpaired, two-tailed Student’s *t* test; for **d**, statistical analysis was performed using one-way ANOVA and Dunnett multiple comparisons test. Data are presented as the means ± S.E.M.; *P* values are shown in the graph. Source data are provided as a Source Data file.

To explore the mechanism by which *Clk4* deficiency led to pathological myocardial hypertrophy and heart failure, we performed a phosphoproteomic assay to screen differentially expressed phosphopeptides/phosphoproteins. A total of 4050 phosphopeptides were included, among which 3361 phosphopeptides in 1326 phosphoproteins were quantifiable (Supplementary Data 1). Using the criteria of a 1.2-fold change and a *P* value < 0.05, a total of 366 significantly differentially expressed phosphopeptides were identified and analyzed, consisting of 190 increased and 176 decreased phosphopeptides (Fig. 4a, Supplementary Fig. 5 and Supplementary Data 1). Heatmaps of the hierarchically clustered upregulated or downregulated phosphoproteomes illustrated the posttranslational modifications induced by *Clk4* deficiency (Fig. 4b). Moreover, Gene Ontology (GO) analysis of the corresponding proteins of these phosphosites indicated that CLK4 was related to the genesis, organization, assembly, and contraction of sarcomeres (Supplementary Fig. 6a). We then performed KEGG pathway analysis and found that the most significantly enriched pathways were the dilated cardiomyopathy pathway; the arrhythmogenic right ventricular cardiomyopathy pathway; the hypertrophic cardiomyopathy pathway; and pathological myocardial hypertrophy-related pathways, such as the notch signaling pathway, adrenergic signaling in cardiomyocytes and the calcium signaling pathway (Supplementary Fig. 6b). Thus, this analysis revealed the phosphoproteome alterations attributable to *Clk4* deficiency, aiding in the discovery of key downstream targets of CLK4.

### CLK4 phosphorylated NEXN at serine 437 to regulate pathological myocardial hypertrophy

To identify potential CLK4 substrates in these quantitative phosphoproteomic data, we used the data filtration parameters of a fold change less than or equal to 60% and a *p* value < 0.01 (*t* test) for further analysis. These analyses uncovered 7 phosphosites on 6 phosphoproteins with reduced phosphorylation levels in *Clk4*-cKO mice, including NEXN, PDHA1, SNW1, DUSP27, ZC3HC1, and NDRG1 (Fig. 4c). Phosphorylation of a protein can modulate its biological activity, subcellular localization, stability and interaction with other proteins^10,11^. We hypothesized that the depressed phosphorylation of these phosphoproteins was associated with the downregulation of their functions. To test this hypothesis, we used specific siRNAs targeting these phosphoproteins to simulate their functional loss due to the reduction of phosphorylated proteins in NRVMs. The siRNA screen showed that only si*Nexn* could mimic the si*Clk4*-induced hypertrophic response in cardiomyocytes, as evidenced by the comparable upregulation of *Nppa* and *Nppb* (Fig. 4d and Supplementary Fig. 2).

NEXN, an F-actin–binding protein that was first characterized in 1998, has been recently identified as a Z-disc protein that is highly abundant in cardiac muscle^12,13^. Multiple mutations in NEXN have been associated with cardiomyopathies^13,14^, highlighting the importance of this protein for cardiac function. However, it is unknown whether NEXN is regulated by phosphorylation and, if so, whether CLK4 is its protein kinase. We, therefore, performed coimmunoprecipitation and revealed that CLK4 and NEXN formed a protein complex (Fig. 4e–g). Moreover, wild-type CLK4 overexpression specifically elevated the serine phosphorylated NEXN (Fig. 4h). To obtain evidence regarding whether CLK4 directly phosphorylates NEXN, we cloned a wild-type fragment of *Nexn* containing potential phosphorylation residues (amino acids 295–671) and an S437A mutant, which were expressed in *E.Coli* and the proteins were affinity-purified for in vitro kinase assay. The results showed that CLK4 phosphorylated the wild-type but not the mutated (S437A) NEXN fragment (Fig. 4i). Interestingly, the phosphorylation site was evolutionarily conserved across species (Supplementary Fig. 7a). We next set to evaluate whether the loss-of CLK4 affected the expression of NEXN by analyzing the mRNA and protein levels of NEXN in the *Clk4*-cKO mouse myocardium. Notably, *Clk4* deficiency did not alter either the mRNA or protein expression of NEXN (Supplementary Fig. 7b, c). However, by using Phos-tag acrylamide gels, we confirmed that the phosphorylated NEXN was reduced in *Clk4*-cKO mouse myocardium (Fig. 4j). Thus, NEXN is a substrate of CLK4, highlighting the potential regulatory role of NEXN in the CLK4-modulated hypertrophic response.

### Restoring NEXN phosphorylation rescued pathological myocardial hypertrophy

**Fig. 5:**
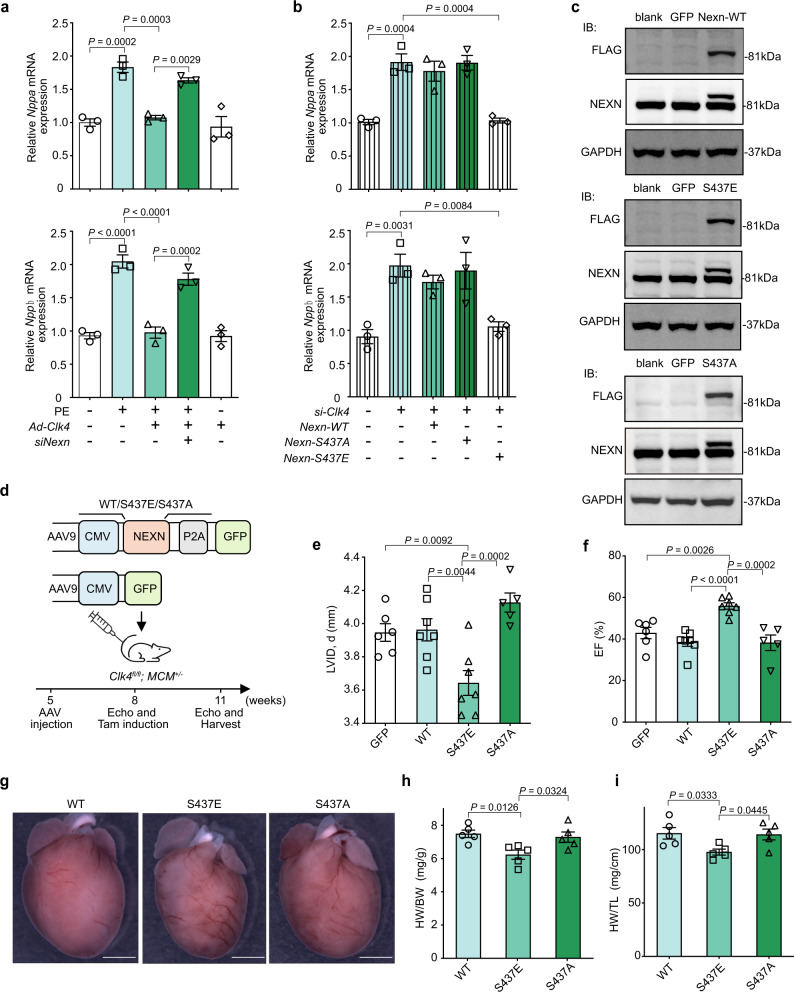
Restoring NEXN phosphorylation corrects the pathological phenotype caused by *Clk4* deficiency in vitro and in vivo. **a** qPCR detection of *Nppa* (upper panel) and *Nppb* (lower panel) in NRVMs challenged with PE alone, Ad-*Clk4* alone, PE + Ad-*Clk4,* or PE + Ad-*Clk4* + *Nexn* siRNA. *n* = 3 biologically independent samples per group. **b** qPCR analysis of *Nppa* (upper panel) and *Nppb* (lower panel) expression in NRVMs transfected with *Clk4* siRNA alone or in combination with *Nexn*-WT, *Nexn*-S437A, or *Nexn*-S437E. WT, wild-type. *n* = 3 biologically independent samples per group. **c** Representative western blots of exogenously expressed and total NEXN, as detected by Flag tag antibody and NEXN antibody respectively, in AAV NEXN-WT (AAV-WT)-, NEXN-S437E (AAV-S437E)- and NEXN-S437A (AAV-S437A)-infected hearts, AAV-GFP and wild-type mice were used as controls. **d** Upper panel, Schematic diagram of the AAV9 vector. Lower panel, protocol for AAV9 administration to *Clk4*-cKO mice. **e**, **f** Summary of echocardiographic data for the LV internal diameter, diastole (LVID, **d**) and LV ejection fraction (EF). *n* = 6 animals for GFP, *n* = 7 animals for WT, *n* = 7 animals for S437E and *n* = 5 animals for S437A. **g** Gross heart images of *Clk4*-cKO mice treated with AAV-WT, AAV-S437E, or AAV-S437A. Scale bar: 2.5 mm. **h** Heart weight-to-body weight ratios (HW/BW). *n* = 5 animals per group. **i** Heart weight-to-tibial length ratios (HW/TL). Data are presented as the means ± S.E.M. *n* = 5 animals per group. For **a**, **b**, **e**, **f**, **h**, and **i**, statistical analyses were performed using one-way ANOVA and Dunnett multiple comparisons test. Data are presented as the means ± S.E.M.; *P* values are shown in each graph. Source data are provided as a Source Data file.

**Fig. 6:**
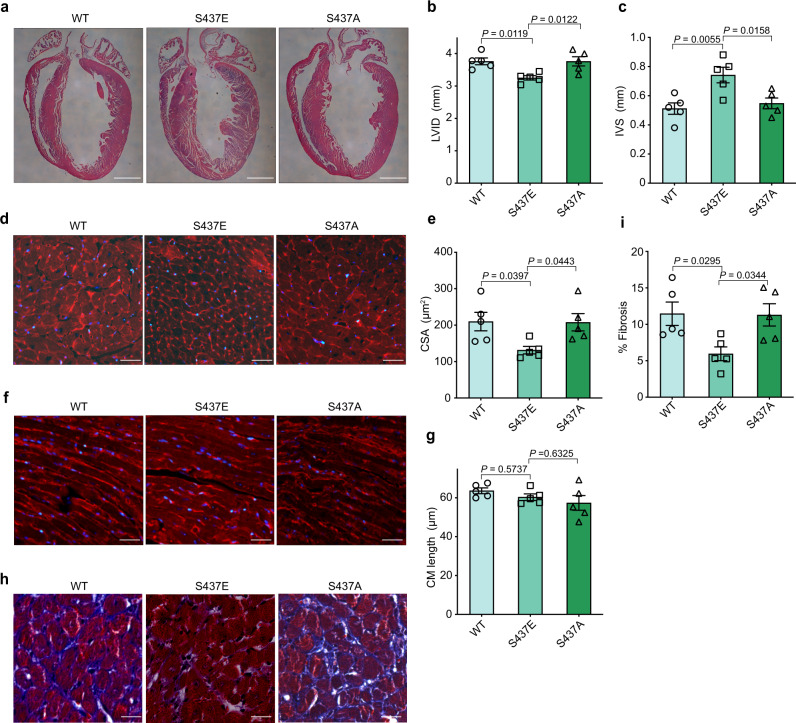
Histological characterization of *Clk4*-cKO hearts with NEXN phosphorylation supplementation. **a** H&E-stained heart sections from AAV-WT, AAV-S437E, and AAV-S437A hearts. Scale bar: 1 mm. **b**, **c** Summary data for LV internal diameter (LVID) and interventricular septum (IVS) thickness. **d**, **e** WGA staining, and quantification of the cardiomyocyte cross-sectional area. Scale bar: 50 μm. **f**, **g** WGA staining, and quantification of the cardiomyocyte length. Scale bar: 25 μm. **h**, **i** Masson’s trichrome staining of heart sections and quantification of myocardial fibrosis. Scale bar: 50 μm. For all panels, *n* = 5 animal per group. All statistical analyses were performed using one-way ANOVA and Dunnett multiple comparisons test. Data are presented as the means ± S.E.M.; *P* values are shown in each graph. Source data are provided as a Source Data file.

To further understand the effect of NEXN on CLK4-regulated myocyte hypertrophy, first, we decided to use an in vitro cardiomyocyte model. On the one hand, cardiomyocytes overexpressing *Clk4* were resistant to phenylephrine (PE)-induced hypertrophy, which was rescued by simultaneous knockdown of *Nexn*. On the other hand, overexpression of a phosphorylation-mimic mutant of NEXN (S437E) but not wild-type NEXN or a phosphorylation-null mutant of NEXN (S437A) inhibited *Clk4* knockdown-induced cardiac hypertrophy (Fig. 5a, b). Likewise, cell size measurements also emphasized the critical role of CLK4-NEXN axis in myocyte hypertrophy regulation (Supplementary Fig. 8). We then sought to analyze the effect of exogenously expressing a phosphorylation-mimic mutant of NEXN (S437E) in *Clk4*-cKO mice. Mice have intravenously injected AAV9 encoding either NEXN-WT (AAV-WT) or mutant NEXNs (AAV-S437E, AAV-S437A), leading to robust overexpression of NEXN in the heart (Fig. 5c, d). As expected, only AAV-S437E reversed the pathological phenotype in *Clk4*-cKO mice, as shown by the reduced LVID, d values, and improved EF values in the AAV-S437E-treated mice compared with mice receiving the AAV-WT or AAV-GFP (Fig. 5e, f and Supplementary Table 6). Morphological improvements were indicated by lower heart weight-to-body weight ratio as well as heart weight-to-tibial length ratio in AAV-S437E-treated *Clk4*-cKO mice (Fig. 5g–i). Further microstructural examinations, including H&E staining, WGA staining, and Masson’s trichrome staining, revealed decreased cardiomyocyte size and attenuated fibrosis in AAV-S437E-treated *Clk4*-cKO mice (Fig. 6a–i). Similarly, upregulation of *Nppa* and *Nppb* due to knockout of *Clk4* was reversed via supplementation with AAV-S437E instead of AAV-WT or the AAV control (Supplementary Fig. 9). Thus, the mechanism by which cardiomyocyte-specific loss-of *Clk4* leads to cardiac hypertrophy and heart failure might involve a decrease in NEXN phosphorylation.

To investigate how sustained NEXN phosphorylation ameliorated the pathological phenotype in *Clk4*-cKO mice. We performed microarray analysis in the *Clk4*-cKO myocardium treated with AAV NEXN-S437E, wherein AAV NEXN-WT served as a control. As demonstrated in Supplementary Fig. 10 and Supplementary Data 2, using the criteria of a 2-fold change and a *P* value < 0.05, a total of 430 significantly differentially expressed mRNAs consisting of 96 increased and 334 decreased mRNAs were identified and analyzed. GO analysis indicated that supplementation of NEXN-S437E was related to calcium and integrin binding, and interstitial remodeling, which are essential processes affecting cardiac function and remodeling. Furthermore, KEGG pathway analysis revealed that the most significantly enriched pathways contain the dilated cardiomyopathy pathway; the hypertrophic cardiomyopathy pathway; and pathological myocardial hypertrophy-related pathways, such as AMPK signaling pathway, PI3k-Akt pathway, and the calcium signaling pathway. These results suggested that NEXN phosphorylation deficiency might underlie cardiac dysfunction in disease settings.

## Discussion

Herein, we reveal that CLK4 acts as a key regulator of pathological cardiac hypertrophy and that its direct substrate NEXN might have a critical role in the process. Four major findings from our in vitro and in vivo experimental models support this conclusion. First, downregulation of *Clk4* led to a hypertrophic phenotype in cardiomyocytes, and overexpression of *Clk4* inhibited phenylephrine-induced hypertrophy. Second, CLK4 expression was significantly reduced in both pressure overload-induced and ISO-induced failing hearts. Third, cardiac-specific *Clk4* knockout contributed to pathological myocardial hypertrophy and heart failure. Finally, *Clk4* deficiency depressed the phosphorylation level of NEXN at serine 437, and restoring NEXN phosphorylation via overexpression of a phosphorylation-mimic mutant of NEXN (S437E) reversed the pathological phenotype resulting from cardiac-specific *Clk4* knockout in mice.

Given that CLKs function mainly by changing the phosphorylation levels of specific targets, we sought to reveal the phosphoproteomic profiles associated with *Clk4* deficiency. Interestingly, our analysis did not identify significantly reduced phosphorylation of SR proteins, which are believed to be downstream targets of CLK4^15–17^, suggesting that a splicing-independent signaling pathway was involved. We identified the phosphopeptides with the most significant reductions in phosphorylation upon CLK4 deletion in myocardial tissue. Among the chosen corresponding phosphoproteins, pyruvate dehydrogenase E1 subunit alpha 1 (PDHA1) ranked first. The PDHA1 catalyzes the reaction that produces acetyl-CoA and CO_2_ from pyruvate, thus facilitating ATP production through glucose. PDHA1 activity is modulated by reversible phosphorylation: phosphorylation of at least one of three specific serine residues of PDHA1 (sites 1, 2, and 3 are S293, S300, and S232, respectively) by pyruvate dehydrogenase kinases (PDKs) inactivates PDHA1, while dephosphorylation of PDHA1 by pyruvate dehydrogenase phosphatase catalytic Subunit 1 (PDP1) restores PDHA1 activity^18–20^. As mentioned above, depression of PDHA1 phosphorylation aids in energy production and therefore seems to be cardioprotective, which is inconsistent with our observations. Additionally, PDHA1 knockdown with siRNA does not stimulate hypertrophy in cardiomyocytes, further suggesting that PDHA1 plays a dispensable role in Clk4 deficiency-induced cardiomyocyte hypertrophy. Meanwhile, the knockdown of *Nexn* can induce a hypertrophic response similar to that of *Clk4* knockdown, underscoring the potential link between NEXN and CLK4.

NEXN, an F-actin–binding protein that was first characterized in 1998, has been recently identified as a Z-disc protein that is highly abundant in cardiac muscle^13^. Multiple mutations in NEXN have been associated with cardiomyopathies, highlighting the importance of this protein for cardiac function^13,14^. Most recently, NEXN was demonstrated to be a new component of junctional membrane complexes (JMCs) required for cardiac T-tubule formation^21^. However, the unique role of NEXN in cardiomyocyte hypertrophy remains to be elucidated. Here, we show that the knockdown of *Nexn* induces cardiac hypertrophy in cardiomyocytes. CLK4 interacts and phosphorylates NEXN at serine 437, and reduced NEXN phosphorylation is associated with cardiac hypertrophy and dysfunction. Most importantly, restoring NEXN phosphorylation corrects the hypertrophic growth and dysfunction induced by knockdown or knockout of *Clk4* in vitro and in vivo. The finding that CLK4 does not affect NEXN mRNA or protein expression suggests that NEXN executes its activity or related interactions in phosphorylated form. Our microarray analysis revealed a set of gene transcripts affected by NEXN phosphorylation, which implies an important role associated with phosphorylation states of NEXN in cardiac pathophysiology. The function of these target genes in cardiac hypertrophy warrants further investigation. We agree that the specific roles of S437 phosphorylation in overall NEXN function are not understood, and further in vivo and in vitro experiments with phosphor-mimetic or phospho-null knock-in mutants of NEXN are warranted to clarify them. Taken together, these findings indicate that *Clk4* deficiency-mediated heart failure may be caused primarily by decreased NEXN phosphorylation.

The present study has several limitations. First, our results obtained in two models of heart failure indicate that the CLK4 protein is downregulated in the myocardium, yet how CLK4 is regulated in these settings remains to be further investigated. Second, our in vivo experiments were mainly conducted in rodents (mice), which have interspecies differences from humans; thus, future research should be performed on human tissue. Third, a phosphosite-specific antibody for detection of phosphorylated NEXN S437 needs to be designed and produced, which will facilitate future functional and mechanical investigation.

In summary, our findings identify CLK4 as a regulator of pathological cardiac hypertrophy and indicate that restoration of CLK4 may act as a potential new therapeutic strategy for heart failure. Moreover, clarifying the importance of CLK4-modulated NEXN phosphorylation in cardiac pathophysiology will shed new light on the function of this Z-disk protein.

## Methods

### Animals

All animal care and experimental protocols were performed in accordance with the 8th edition of the Guide for the Care and Use of Laboratory Animals and were approved by the Institutional Animal Care and Use Committee of the Tongji University School of Medicine (Shanghai, China). Isoflurane (4%) mixed with oxygen (100%, 1 L/min) was used as inhalable anesthesia in mice prior to harvesting the heart. The mouse was euthanized by cervical dislocation after harvest was completed.

### Isolation and transfection of NRVM

Cardiomyocytes were prepared from the hearts of 1- to 2-day-old (newborn) Sprague–Dawley rats as previously described^22^. Briefly, all pups were euthanized via cervical dislocation and sterilized, and the hearts were excised. Only the ventricles were collected. The ventricles were minced and deposited into an enzyme solution containing trypsin (0.125 mg/ml, Gibco), collagenase type IV (0.4 mg/ml, Worthington), and DNase II (10 mg/ml, Sigma). Serial digestions were performed in a water bath at 37 °C with shaking at 80–86 rpm. Cell suspensions of each digestion were collected in Dulbecco’s modified Eagle’s medium (DMEM, Gibco) supplemented with 10% fetal bovine serum (FBS, Gibco). After all digestions, the cell suspensions in FBS were pooled and centrifuged for 5 min at 100 × *g*. The pellets were then resuspended in DMEM supplemented with 10% FBS and 100 mM 5-bromodeoxyuridine (BrdU; Sigma) plating medium and cultured on a 10-cm dish at 37 °C in 5% CO_2_ for 2 h. The resultant supernatants represented enriched neonatal cardiomyocytes, which were collected and then plated onto 1% gelatin (Sigma)-coated plates. The next day, the cells were washed and the medium was switched to DMEM supplemented with 2% FBS, 100 mM BrdU and 1% penicillin/streptomycin.

On day 3, the cardiomyocytes were transfected with the indicated siRNAs at 50 nM final concentration using Lipofectamine RNAiMAX (Invitrogen). Cells transfected with a scrambled siRNA were used as controls. All siRNA sequences are listed in Supplementary Table 1. The cells were cultured for an additional 48 h and then analyzed by qPCR and immunofluorescence.

### Culture and transfection of hiPSC-CMs

The hiPSC-CMs were purchased from Help Stem Cell Innovation (Nanjing, China), and were approved for research use by the Ministry of Science and Technology of the People’s Republic of China ([2021]-BC0022). Cell culture and maintenance were performed according to the protocol of the manufacturer. hiPSC-CMs were transfected with Lipofectamine RNAiMAX (Invitrogen) as mentioned above.

### Immunofluorescence staining and evaluation of cardiomyocyte hypertrophy

Immunofluorescence staining of NRVMs and hiPSC-CMs was carried out by following standard protocols. Briefly, NRVMs or hiPSC-CMs were fixed in 4% paraformaldehyde, permeabilized with 0.1% triton X-100, and blocked with 0.1% bovine serum albumin (BSA). Cells were incubated overnight with anti-cTNT (1:200, Abcam, ab8295) at 4 °C, and then for 1 h with an Alexa Fluor 488-conjugated anti-mouse secondary antibody (1:500, Invitrogen, A11001). Nuclei were counterstained with DAPI (Sigma, D9542). Cells were visualized using a Leica DMI3000 B fluorescence microscope (Leica Microsystems, Inc.). Cell size (> 80 cells per group) was quantified using ImageJ (NIH).

### qPCR and western blot analyses

Total RNA was isolated from cells or tissue samples using TRIzol Reagent (Ambion) according to the manufacturer’s protocol. For qPCR, 500 ng of RNA sample was reverse-transcribed into cDNA by using random hexamers and MMLV reverse transcriptase (Takara) in a 10-μl reaction system. In each analysis, a 0.1-μl cDNA pool was used for qPCR. qPCR was then performed using SYBR Green Real-time PCR Master Mix (Toyobo, QPK-201) on a QuantStudio 6 Real-Time PCR System (Applied Biosystems). The housekeeping gene *Gapdh* was used for normalization. The qPCR primers used in this study are listed in Supplementary Table 2.

For Western blot analyses, tissue homogenates or cell extracts were cleared by centrifugation at 12,000 × *g* for 20 min. The samples were subsequently analyzed by sodium dodecyl-sulfate polyacrylamide gel electrophoresis (SDS-PAGE) and transferred to polyvinylidene difluoride (PVDF) membranes, which were blocked with 5% nonfat dry milk in TBS + 0.1% Tween 20 (TBST) and incubated with anti-CLK4 (1:500, Abcam, ab104321), anti-GAPDH (1:8000, Proteintech, #60004-1-Ig), anti-NEXN (1:500, Abcam, ab233235), anti-flag (1:5000, Sigma, F7425), anti-myc (1:1000, Cell Signaling Technology, #2276), and anti-Phosphoserine (1:1000, Sigma, #05-1000X) antibodies overnight at 4 °C. The membranes were washed three times with TBST, and incubated with a secondary antibody conjugated with Alexa Fluor 680/800 (1:10000, Invitrogen, A32729/A32735). Specific protein bands were visualized using an Odyssey imager. The Western blot bands were quantified by densitometry using ImageJ software (NIH).

### Generation of *Clk4*-cKO mice

*Clk4*-cKO mice were generated using a CRISPR/Cas9 system. Two sgRNAs (5′ sgRNA 5′-GCAACGATGCTGTATCATTT-TGG-3′ and 3′ sgRNA 5′-CTATATAGTGAAATTAAGGC-AGG-3′) with recognition sites on introns 2 and 10 of the *Clk4* gene and a donor vector that was homologous with the targeted gene and contained two loxP sequences were designed and constructed on a C57B/6 J background. The in vitro-synthesized sgRNA, *Cas9* mRNA, and donor vectors were injected into mouse zygotes, which were then transferred into pseudopregnant mice. Neonatal mutant mice were identified by genotyping and sequencing (Supplementary Fig. 1 and Supplementary Table 3). The resulting *Clk4*^*fl/fl*^ mice were crossed with mice carrying the *α-MHC-MerCreMer* (MCM) transgene (available from Jackson Laboratory, stock no. 005657) to generate inducible *Clk4* cardiac-specific knockout (*α-MHC-MerCreMer*; *Clk4*^*fl/fl*^) mice. For temporal deletion of *Clk4* using the MCM model, male mice 6–8 weeks of age were injected intraperitoneally with tamoxifen (25 mg/kg per day) for five consecutive days. Both *Clk4*^*fl/fl*^ and MCM mice were used as controls. The cardiac phenotype of the mice was analyzed 3 weeks after the first tamoxifen injection^23,24^.

### Heart failure models

Both pressure overload-induced and agonist-induced heart failure models were used. For the pressure overload model, 7-week-old male were anesthetized with 3% isoflurane. After intubation, the animals were maintained on 1% isoflurane and 1 L/min oxygen. We applied a method that was less invasive than the most common method to induce left transverse aortic constriction, as previously described^25^. In brief, the aortic arch was accessed through a midline incision in the anterior neck instead of via thoracotomy, and a 6.0 silk suture was placed around the transverse aorta between the brachiocephalic trunk and the left common carotid artery. The suture was tightened around a blunt 27-gauge needle placed adjacent to the aorta. The needle was then removed, and the chest wall and skin were closed. In the sham operation, the entire procedure was identical except for the ligation of the aorta.

For the agonist-induced mouse model, ISO was administered via subcutaneous injection (150 mg/kg/day) for 7 days as previously described^26^. Over the ensuing 2 months, echocardiography was performed every 2 weeks to evaluate cardiac function. Mice with an EF < 40% were assigned to the heart failure group and were sacrificed along with the controls. The hearts were immediately dissected and frozen in liquid nitrogen.

### Measurement of cardiac function by echocardiography

Echocardiography was performed on mice using a Vevo 2100 Imaging System (VisualSonics) with a 30-MHz MicroScan transducer (model MS-400). Mice were anesthetized with isoflurane (3% isoflurane for induction and 1% for maintenance). The heart rate and left ventricular (LV) dimensions, including diastolic and systolic wall thicknesses and LV end-diastolic and end-systolic chamber dimensions, were measured from 2D short-axis views under M-mode tracings at the level of the papillary muscle. The LV mass and functional parameters, such as the percentage of EF and the fractional shortening, were calculated based on the above primary measurements with accompanying software.

### Histological analysis

Animals were anesthetized with 4% isoflurane and then sacrificed by injection of 10% KCl to stop the heart at diastole. The hearts were excised, rinsed with PBS, and fixed in 4% PFA (pH 8.0) overnight. After dehydration through a series of ethanol baths, the samples were embedded in paraffin and further processed for histology. For routine histological examination with a light microscope, 5-μm sections were stained with H&E and Masson’s trichrome in accordance with standard procedures to visualize the regular morphology and the extent of fibrosis. For fibrosis detection, we mainly referred to the methodological descriptions of two studies^27,28^, and 10 focal regions from > 15 sections obtained from serial sectioning of each heart (apex to base) were calculated. For cardiomyocyte CSA and length measurements, sections were stained with FITC-conjugated WGA (Invitrogen, W7024), and then measured by ImageJ (> 50 cells per animal).

### Transmission electron microscopy

LV samples were carefully cut into 2-mm^3^ pieces and fixed in 2.5% glutaraldehyde (Sigma, G5882) in 0.1 M sodium phosphate buffer (pH 7.4) for 2 h. Following buffer washes, the samples were fixed for an additional 2 h at room temperature with 1% osmium tetroxide. Then, the samples were dehydrated in an ascending acetone series, embedded in epoxy resin, and polymerized at 60 °C for 24 h. The prepared tissues were sectioned to 70–100 nm, stained with uranyl acetate and lead citrate, observed under a JEM-1230 transmission electron microscope, and photographed.

### Phosphoproteomic analysis

Analysis of protein phosphorylation in ventricular tissues extracted from both the *Clk4*-cKO and MCM control groups was performed using tandem mass tags (TMTs) from Thermo Fisher Scientific. Briefly, protein from the hearts of *Clk4*-cKO mice and their MCM littermate controls were solubilized, digested with LysC and trypsin, labeled with TMT-6 plex reagents, and mixed in batches of six to enable direct comparisons between Clk4-cKO (*n* = 3) and MCM (*n* = 3) mice. Phosphopeptides were enriched from the samples using titanium dioxide (TiO_2_) beads as previously described^29^ and then subjected to LC-MS/MS analysis using an Orbitrap Fusion Lumos Tribrid mass spectrometer interfaced with an Easy-nLC 1200 liquid chromatography system (both from Thermo). Peptides were trapped onto an Acclaim PepMap 100 C18 trap column (100 μm × 2 cm, Thermo) and eluted onto an analytical column (Acclaim PepMap RSLC 75 μm × 15 cm, Thermo) at a flow rate of 300 nl/min using 0.1% formic acid in water as solvent A and 80% acetonitrile and 0.1% formic acid as solvent B. Each sample was run on a 90-minute gradient.

Full MS scans were recorded at 60,000 resolution in the m/z range 350–1800 with automatic gain control (AGC) target ion intensity of 4 × 10^5^. The ten most intense peaks in MS were fragmented with higher-energy collisional dissociation with a normalized collision energy of 36. MS/MS scans were obtained at a resolution of 15,000 with an AGC target value of 1 × 10^5^ and a maximum injection time of 80 ms. The dynamic exclusion time was set to 60 sec.

MS/MS spectra were searched against the Mus musculus proteome database (UP000000589) using Proteome DiscovererTM V.2.2 (Thermo, USA), and subsequent statistical analysis was performed using Microsoft Excel. The global false discovery rate was set to 0.01, and only proteins identified with a minimum of 2 unique peptides were considered. Phosphorylation sites were identified using the following parameters: sample type, TMT 6 plex (peptide labeled); Cys alkylation, iodoacetamide; variable modification, oxidation (M), acetyl (protein N-term), phospho (STY); digestion, trypsin; instrument, Orbitrap Fusion. GO annotation (http://www.ebi.ac.uk/GOA/) was applied to determine the protein categories that were enriched. Kyoto Encyclopedia of Genes and Genomes (KEGG) pathway mapping of the phosphoproteins was performed using the KEGG Automatic Annotation Server, a KEGG online tool. Only categories with a Fisher’s corrected *P* value < 0.05 were considered significantly enriched.

### Microarrays

We performed microarray analysis using Agilent SurePrint G3 Mouse Gene Expression v2 8x60K Microarray (Design ID: 074809) according to the manufacturer’s protocol.

### Coimmunoprecipitation assay

NRVMs were transiently transfected with plasmids encoding Flag-tagged mouse CLK4 and myc-tagged mouse NEXN using Lipofectamine 3000 Transfection Reagent (Thermo, L3000001). Cells were harvested 48 h after transfection in lysis buffer composed of PBS containing 0.5% Triton X-100, 1 mM EDTA, 1 mM PMSF, and complete protease inhibitors (Roche, 11697498001). After brief sonication and removal of cellular debris by centrifugation, flag-tagged CLK4 proteins were precipitated with anti-flag antibodies and protein A/G beads and analyzed by Western blot with anti-myc antibodies.

### Plasmids

Plasmids were constructed with a method based on restriction enzyme digestion. Mouse *Clk4* and *Nexn* cDNAs from cardiac tissue were amplified using PrimeSTAR Max DNA Polymerase (Takara, R045A) and cloned with a flag or a myc tag into the PCMV-C-HA plasmid (Beyotime, D2639). Plasmids with the S437A or S437E mutation of mouse NEXN were generated using a Mut Express II Fast Mutagenesis Kit V2 (Vazyme, C113). The plasmid PGEX-6p-1 (GE Healthcare) was used for the cloning of GST fusion genes.

### Generation and administration of AAV (serotype 9) and adenovirus

The adenoviral vector pAdM-FH-GFP-Clk4 (wild-type) and a control vector (Ad-Ctrl) were obtained from Vigene Biosciences (Rockville, MD, USA). To achieve in vivo overexpression of NEXN-WT, NEXN-S437E, or NEXN-S437A, mouse *Nexn*-WT, *Nexn*-S437E or *Nexn*-S437A was subcloned into the pAV-CMV-intron-P2A-GFP plasmid. AAV9 vectors for expression of NEXN-WT, NEXN-S437E or NEXN-S437A were generated by cotransfection of a pAV-CMV-intron-P2A-GFP expression plasmid, a Rep-Cap plasmid and a pHelper plasmid in Hek293 cells. AAV was purified and concentrated by gradient centrifugation. The AAV9 titer was determined by qPCR. The AAV9 virus (1 × 10^12^ viral genomes per animal) was intravenously injected into 5-week-old *Clk4*-cKO mice and MCM control littermates.

### Protein purification

The *Clk4*-WT expression construct was transfected into HEK293T cells using Lipofectamine 3000. After culturing for 48 h, the cells were harvested and lysed in lysis buffer containing 50 mM Tris, pH 7.4, 500 mM NaCl, 1% NP40, 1 mM PMSF, 1× protease inhibitors without EDTA (Roche). The recombinant protein was purified with FLAG M2 affinity gel (Sigma) according to the manufacturer’s instructions. The eluted recombinant protein was dialyzed with storage buffer (20 mM HEPES, pH 7.4, 50 mM NaCl, and 50% glycerol) for 4 h, at 4 °C, and then stored at −20 °C. The homogeneity of the eluted protein was determined on SDS-PAGE followed by Coomassie blue staining and western blot using an anti-flag antibody.

For purification of GST-tagged proteins, plasmids were transfected into *E. coli* strain BL21 (DE3), induced to express desired proteins by isopropyl-1-thio-beta (β)-d-galactopyranoside (IPTG) and purified to homogeneity from crude lysates using glutathione-Sepharose beads (Smart Life Sciences, SA010010) according to the manufacturer’s protocol. Briefly, protein expression was induced by adding 1 mM IPTG to the cultures. The bacteria were collected by centrifugation, resuspended in PBS, and lysed by sonication. After centrifugation at 15,000 × *g* for 15 min, the supernatant was incubated with Glutathione-Sepharose beads for 1 h. The beads were washed with PBS three times, and the recombinant protein was eluted with PBS containing 20 mM reduced glutathione. The proteins were dialyzed against PBS and stored at −80 °C until use.

### In vitro kinase assay

Cell-free kinase assay was performed in kinase buffer (CST, 9802 S) containing 25 mM Tris-HCl (pH 7.5), 5 mM β-glycerophosphate, 2 mM dithiothreitol, 0.1 mM Na_3_VO_4_, and 10 mM MgCl_2_. The CLK4 protein was incubated with C-NEXN (amino acids 295–671) or C-NEXN-S437A on ice for 10 min and then exposed to 1 mM ATP (CST, 9804) for 30 min at 30 °C. The reactions were stopped by adding SDS loading buffer. The samples were boiled for 5 min and separated on SDS-PAGE. Commercial antibodies against phosphorylated serine and NEXN were used for western blot analysis^30^.

### Phos-tag SDS-PAGE

Phos-tag analysis was performed according to the manufacturer’s instructions. Heart tissue lysates were analyzed. Samples were separated on 8% SDS-PAGE with 50 μM Phos-binding reagent acrylamide (APExBIO, F4002) and transferred onto PVDF membranes for western blot analyses.

### Statistics and reproducibility

The data are presented as the means ± S.E.M. Unpaired, two-tailed Student’s *t* test or one-way analysis of variance with Dunnett multiple comparisons test was performed as indicated to determine significant differences. All statistical analyses were performed using GraphPad Prism software. *P* < 0.05 was considered statistically significant. The representative data shown as western blot photograph and microscopy images were obtained from at least three independent experiments or at least three mice per group (for Figs. 1b, d; 2c; 3a; 4e; 5c; 6a and Supplementary Figs. 1b; 2b, d, and 7c).

### Reporting summary

Further information on research design is available in the Nature Research Reporting Summary linked to this article.

## Supplementary information

Supplementary information

Reporting Summary

Description of Additional Supplementary Files

Supplementary Data 1

Supplementary Data 2

## Data Availability

The data supporting the findings from this study are available within the manuscript and its supplementary information. The raw MS data generated in this study have been deposited to the ProteomeXchange Consortium via the PRIDE partner repository under accession code PXD029682. The reference number for the mouse Swiss-Prot database used in this study is UP000000589. Microarray data have been deposited to the GEO database with the GEO accession number GSE188360. Source data are provided with this paper.

## Source data

Source data
