# Supplementary material for: CDC-like kinase 4 deficiency contributes to pathological cardiac hypertrophy by modulating NEXN phosphorylation
Source: Nat Commun. 2022 Jul 30;13:4433. doi: 10.1038/s41467-022-31996-9 (PMC9338968; doi:10.1038/s41467-022-31996-9)
Supplement: Supplementary file 1 — Supplementary information [file 41467_2022_31996_MOESM1_ESM.pdf]

# Supplementary Information

## CDC-like kinase 4 deficiency contributes to pathological cardiac hypertrophy by modulating NEXN phosphorylation

**Table of contents:**

Supplementary Fig. 1

Supplementary Fig. 2

Supplementary Fig. 3

Supplementary Fig. 4

Supplementary Fig. 5

Supplementary Fig. 6

Supplementary Fig. 7

Supplementary Fig. 8

Supplementary Fig. 9

Supplementary Fig. 10

Supplementary Table 1

Supplementary Table 2

Supplementary Table 3

Supplementary Table 4

Supplementary Table 5

Supplementary Table 6

Supplementary Fig. 11 (Uncropped images from Western blots)

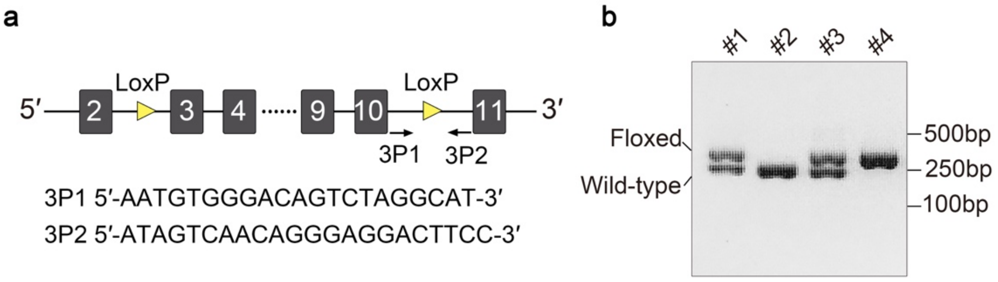


**Supplementary Fig. 1** Genotyping of floxed *Clk4* mice. **a** Genotyping strategy for the floxed *Clk4* allele. PCR primers were designed to flank the 3’ loxP site. **b** Representative gel analysis of PCR products of four mice to separate the 306 bp loxP amplicon from the 210 bp wild-type amplicon.

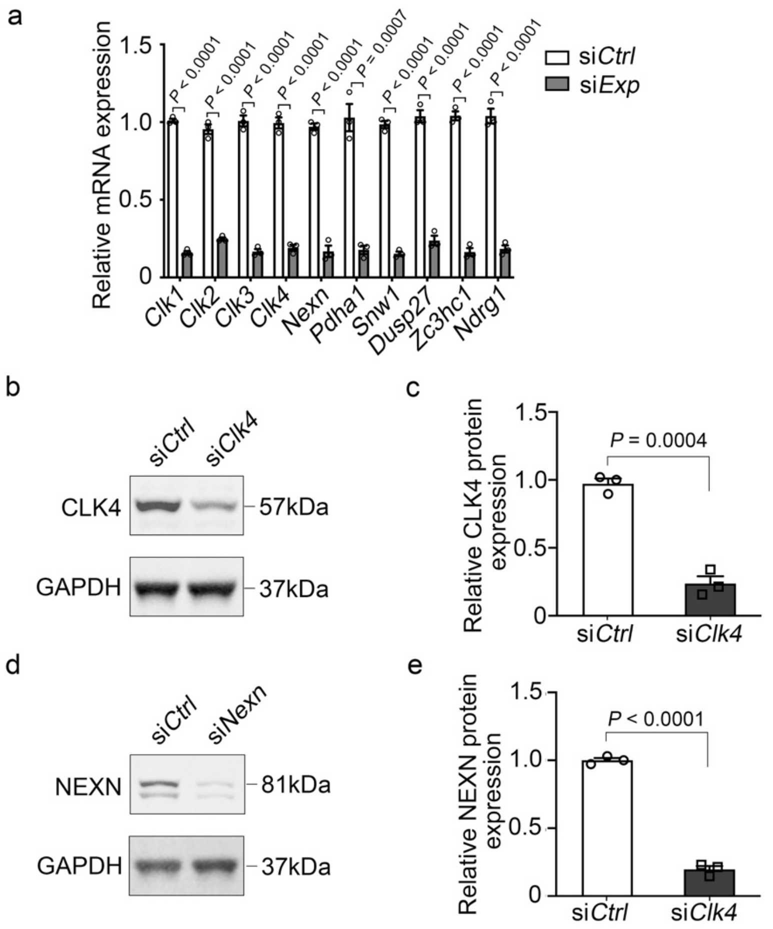


**Supplementary Fig. 2** Validation of the knockdown efficiency of siRNAs using qPCR and Western blotting. Neonatal rat ventricular myocytes (NRVMs) were transfected with siRNAs targeting *Clk1, Clk2, Clk3, Clk4, Nexn, Pdha1, Snw1, Dusp27, Zc3hc1,* and *Ndrg1*. **a** qPCR detection of the mRNA expression of the siRNA-targeted genes. **b-e** Representative Western blots and quantification of CLK4 and NEXN protein expression. Ctrl, control; Exp, experiment. For panel **a**, **c** and **e**, n = 3 biologically independent samples per group. All statistical analyses were performed using unpaired, two-tailed Student's t test. Data are presented as the means ± S.E.M.; *P*-values are shown in each graph. Source data are provided as a Source Data file.

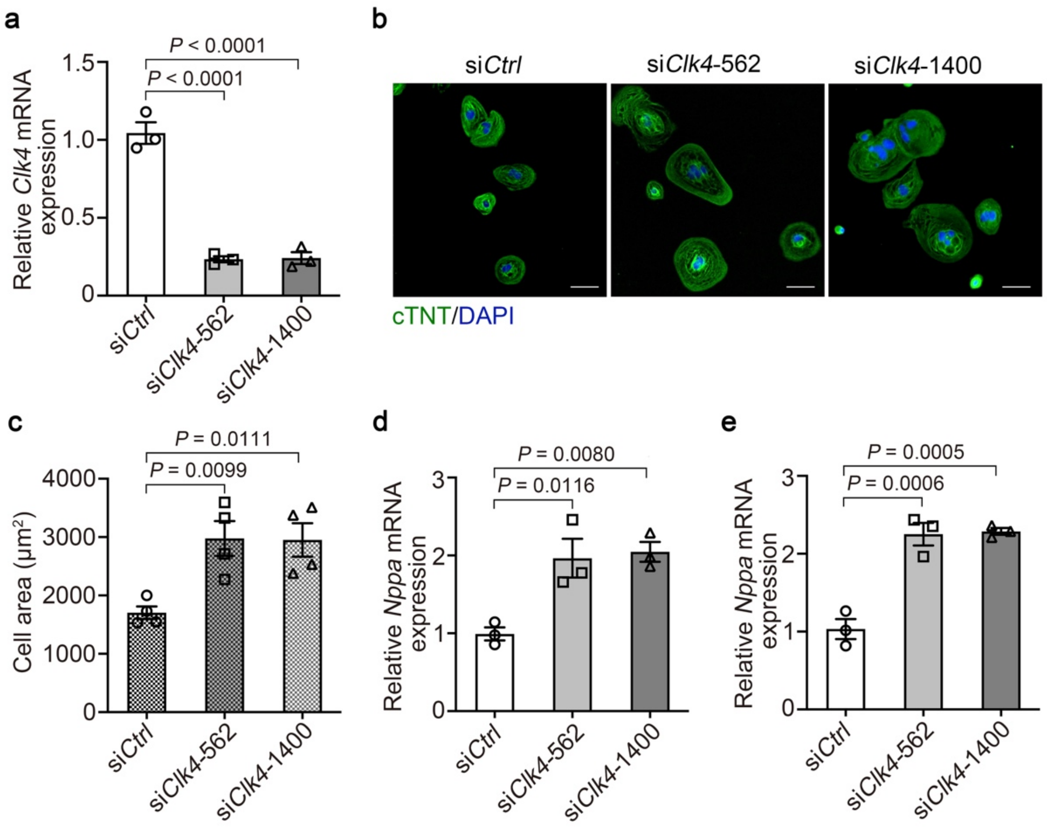


**Supplementary Fig. 3** Knockdown of *Clk4* leads to cardiac hypertrophy in human-induced pluripotent stem cell-derived cardiomyocytes (hiPSC-CMs). **a** qPCR detection of the expression of *Clk4* mRNA in hiPSC-CMs transfected with control or *Clk4* siRNAs. **b**, **c** Representative image of NRVMs transfected with the control or *Clk4* siRNA for 48 h with cell area quantification. Scale bar: 50 μm. **d**, **e** qPCR detection of the expression of *Nppa* and *Nppb* in hiPSC-CMs transfected with control or *Clk4* siRNAs. For panel **a**, **d** and **e**, n = 3 biologically independent samples per group; for panel **c**, n = 4 biologically independent samples per group. All statistical analyses were performed using one-way ANOVA and Dunnett multiple comparisons test. Data are presented as the means ± S.E.M.; *P*-values are shown in each graph. Source data are provided as a Source Data file.

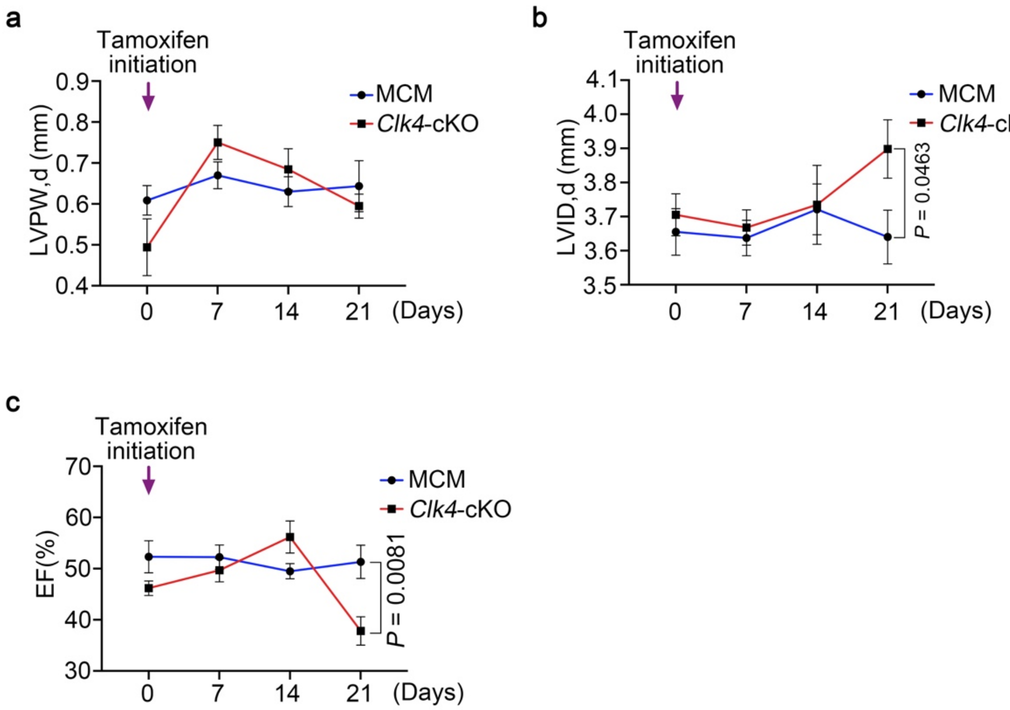


**Supplementary Fig. 4** Cardiac-specific *Clk4* knockout (*Clk4*-cKO) mice develop heart failure gradually. *Clk4*-cKO mice and littermates aged 6 weeks were used in the experiments. *Clk4* knockout was achieved by tamoxifen injection, and an echocardiographic analysis was performed every week. **a-c** Summary data for the left ventricular posterior wall, diastole (LVPW, d), left ventricular internal diameter, diastole (LVID, d) and left ventricular ejection fraction (EF). n = 6-8 animals for MCM and n = 7-8 animals for *Clk4*-cKO. All statistical analyses were performed using unpaired, two-tailed Student's t test. Data are presented as the means ± S.E.M.; *P*-values are shown in the graph. Source data are provided as a Source Data file.

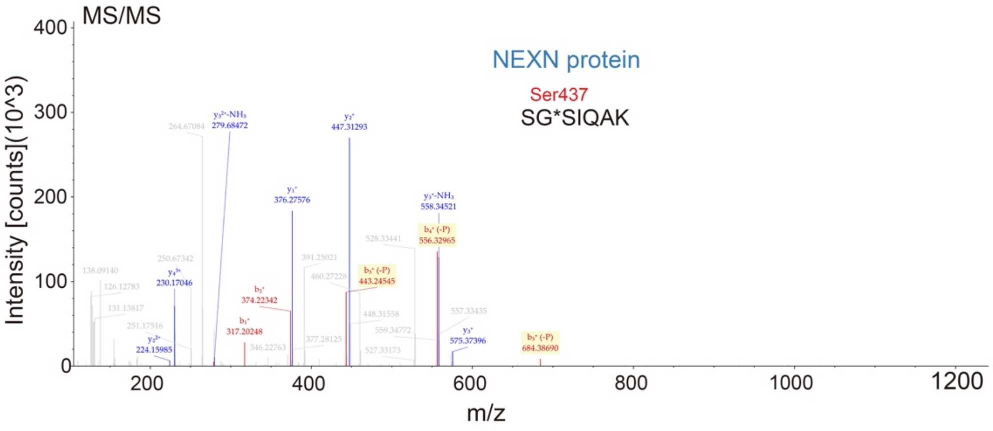


**Supplementary Fig. 5** MS/MS spectra of NEXN phosphopeptide SGSIQAK. Representative tandem mass spectra of NEXN phosphopeptide SHS*SSQFR identified with reduced abundance in the *Clk4*-cKO myocardium. The asterisk denotes phosphorylation at Ser437.

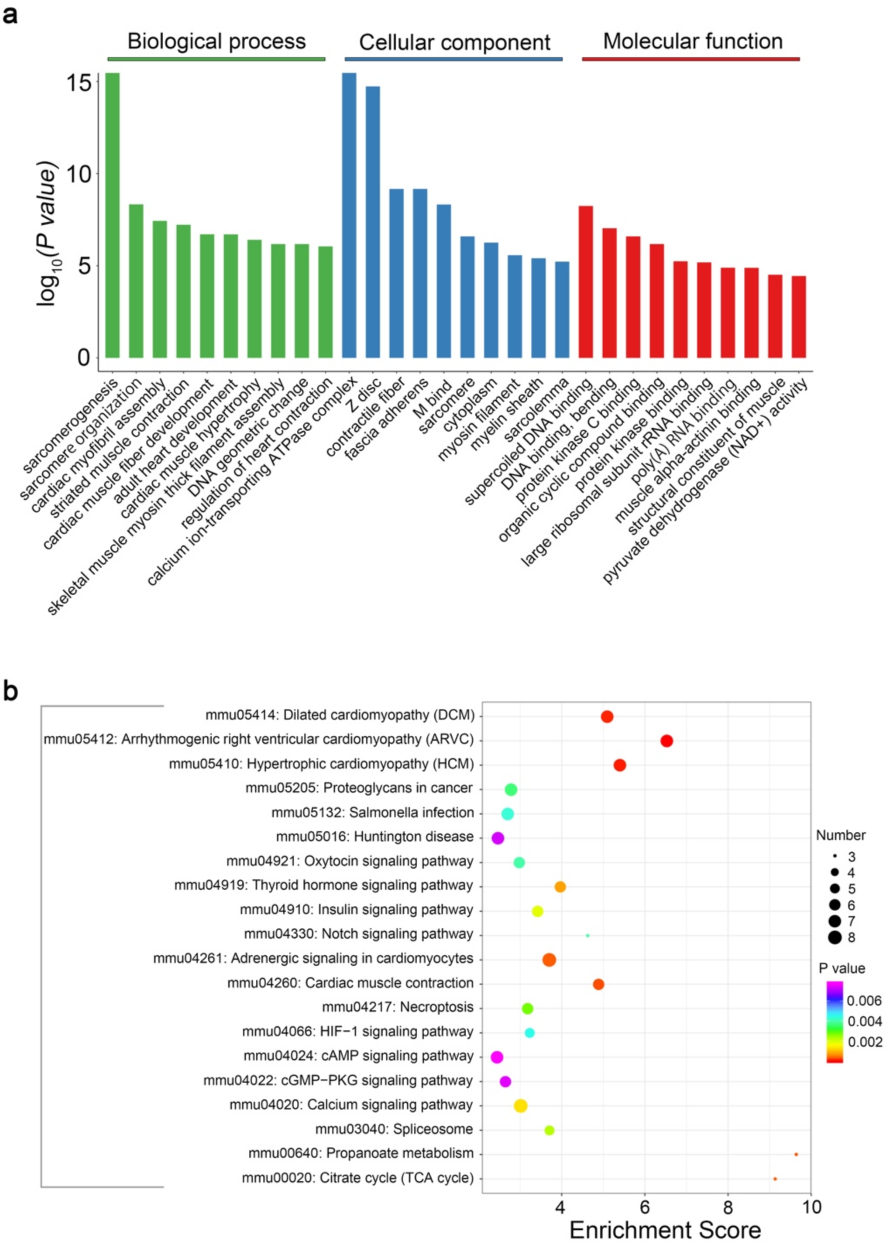


**Supplementary Fig. 6** GO and KEGG enrichment analyses of genes related to *Clk4* knockout. **a** Top ten GO terms in each of the three categories, molecular function (green), cellular component (blue) and biological function (red), for the differences in the phosphoproteome between *Clk4*-cKO and MCM. **b** Top twenty signaling pathways in the *Clk4*-cKO/MCM comparison according to KEGG enrichment analysis. n = 3 animals per group.

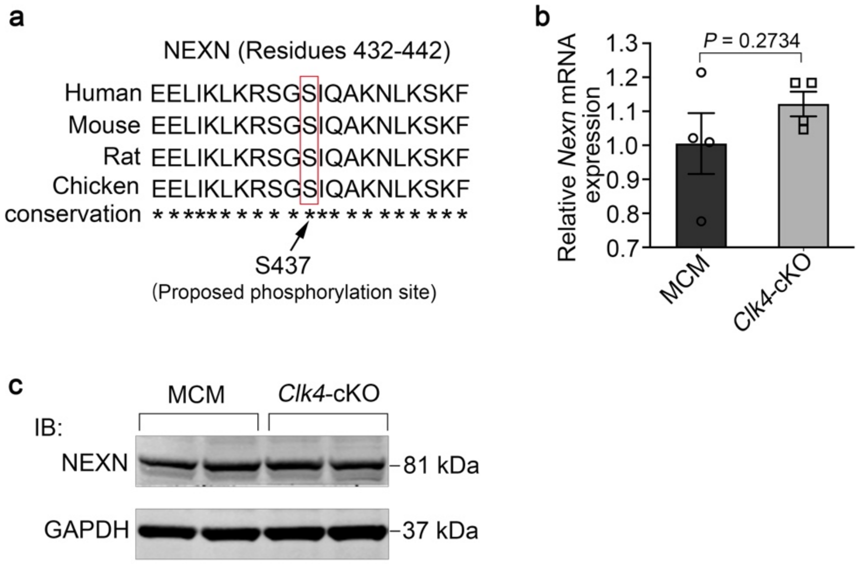


**Supplementary Fig. 7** Clk4 deficiency does not alter either the mRNA or protein expression of NEXN. **a** Sequence alignment revealed that the phosphorylation site was evolutionarily conserved across species. **b**, **c** qPCR and Western blot analysis of mRNA and protein expression of mouse NEXN in *Clk4*-cKO and MCM control hearts. GAPDH served as control. For panel **b**, n = 4 animals per group. Statistical analysis was performed using unpaired, two-tailed Student's t test. Data are presented as the means ± S.E.M.; *P*-values are shown in the graph. Source data are provided as a Source Data file.

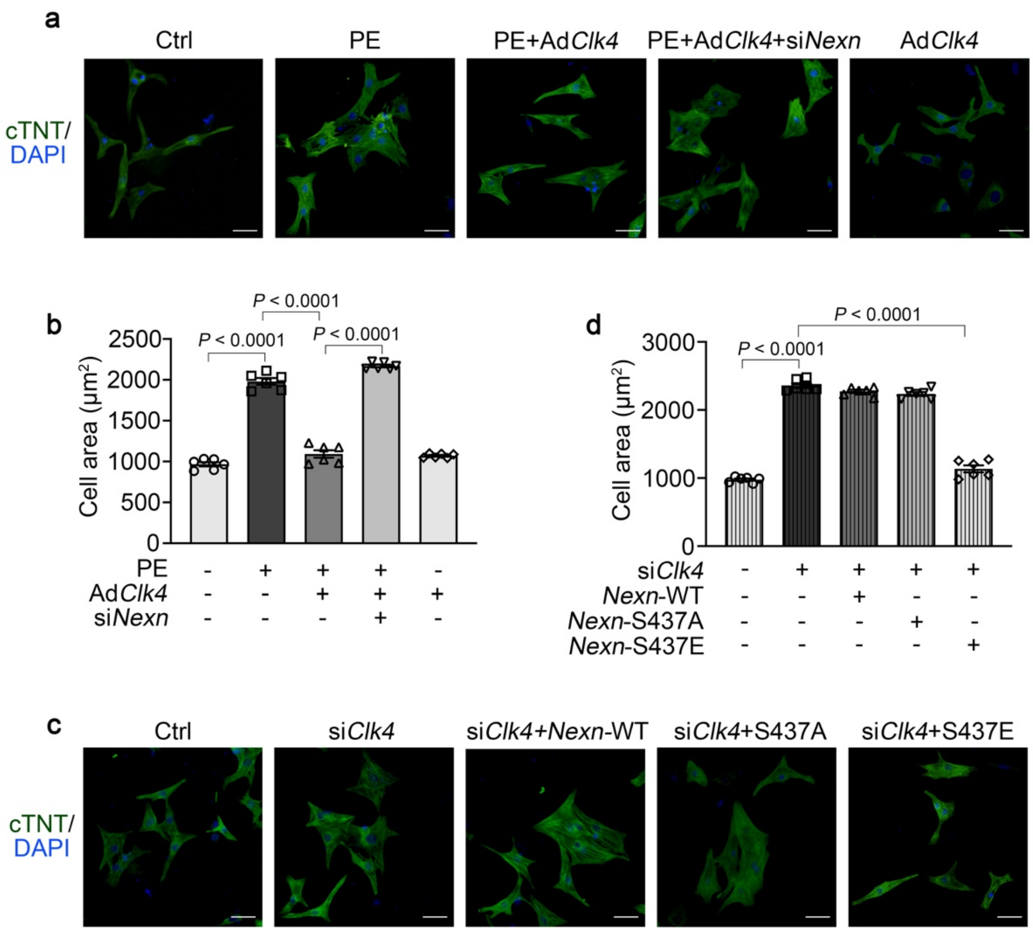


**Supplementary Fig. 8** CLK4 modulates cardiomyocyte hypertrophy through regulating NEXN phosphorylation. **a**, **b** Representative immunostaining images of NRVMs challenged with PE alone, Ad-*Clk4* alone, PE + Ad-*Clk4* or PE + Ad-*Clk4* + *Nexn* siRNA with cell area quantification. Scale bar: 50 µm. **c**, **d** Representative immunostaining images of NRVMs transfected with *Clk4* siRNA alone or in combination with NEXN-WT, NEXN-S437A or NEXN-S437E. Scale bar: 50 µm. For panel **a**-**d**, n = 6 biologically independent samples per group. All statistical analyses were performed using one-way ANOVA and Dunnett multiple comparisons test. Data are presented as the means ± S.E.M.; *P*-values are shown in each graph. Source data are provided as a Source Data file.

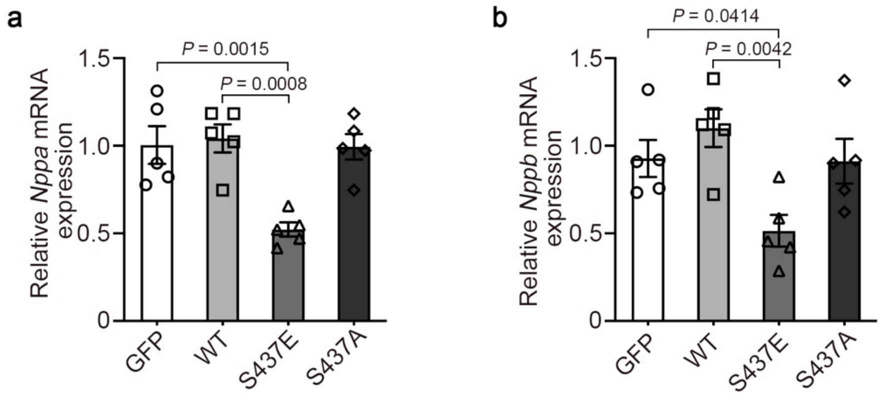


**Supplementary Fig. 9** Restoration of NEXN phosphorylation reduces the expression of hypertrophic markers in *Clk4*-cKO mice. **a**, **b** qPCR analysis of *Nppa* (**a**) and *Nppb* (**b**) expression in *Clk4*-cKO hearts after intravenous administration of AAV-GFP, AAV-WT, AAV-S437E or AAV-S437A. n = 5 animals per group. All statistical analyses were performed using one-way ANOVA and Dunnett multiple comparisons test. Data are presented as the means ± S.E.M.; *P*-values are shown in each graph. Source data are provided as a Source Data file.

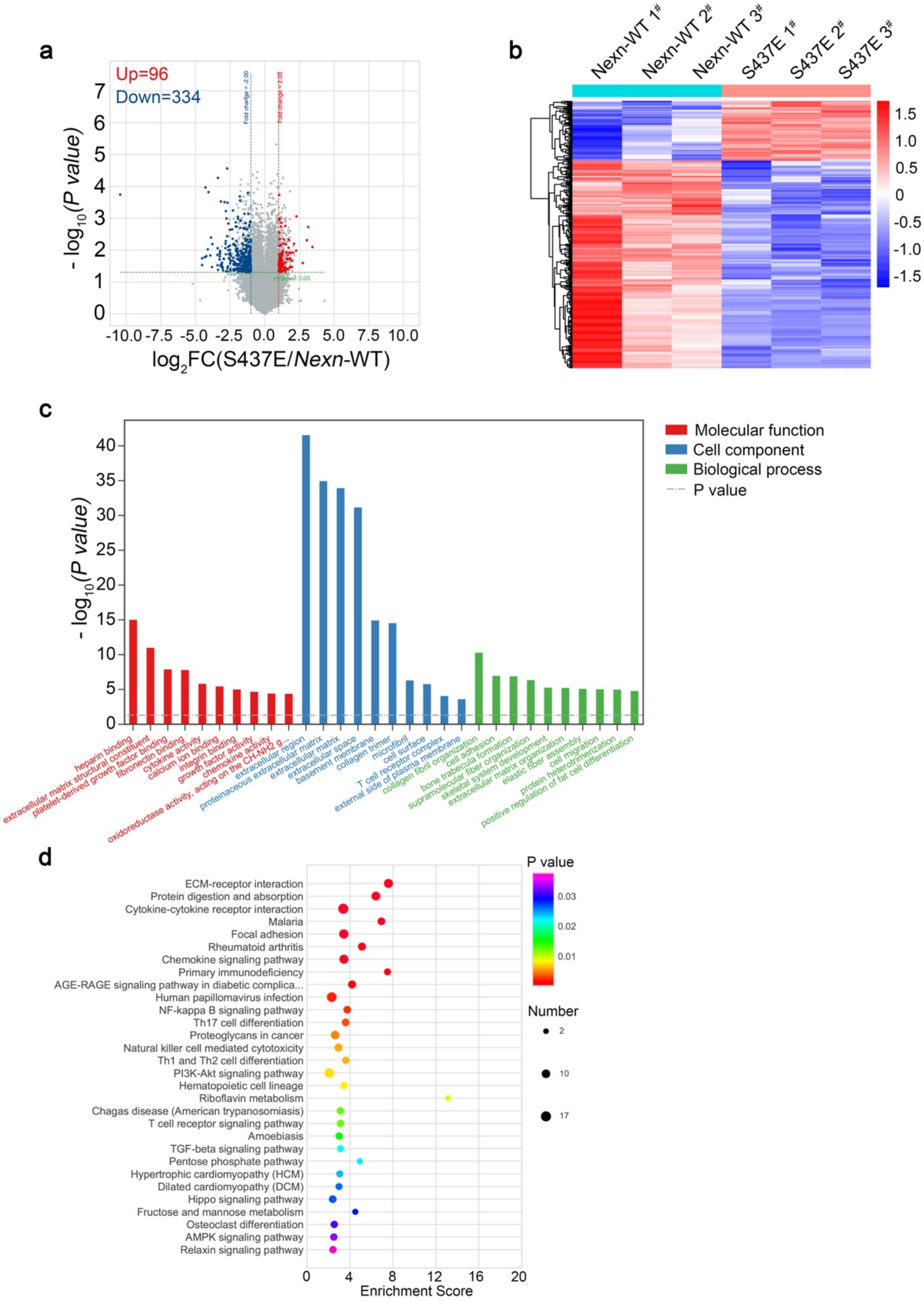

**Supplementary Fig. 10** Microarray analysis of *Clk4*-cKO hearts supplemented with NEXN-S437E overexpression. **a** Volcano plots showing changes in mRNAs in AAV-S437E-compared with AAV-WT-treated in *Clk4*-cKO mice, as identified by RNA microarray analysis. The red dots are mRNAs with fold-changes (S437E/WT ratios) > 2 ($P$ < 0.05, unpaired, two-tailed Student's t test), and the blue dots are those with fold-changes < 1/2 ($P$ <

0.05, unpaired, two-tailed Student's t test). **b** Hierarchical cluster analysis of the specified mRNAs. **c** Top ten GO terms in each of the three categories, molecular function (red), cellular component (blue) and biological function (green), for the difference between the S437E and WT NEXN. **d** Top 30 signaling pathways in the S437E/WT comparison according to KEGG enrichment analysis. n = 3 animals per group.

**Supplementary Table 1** The target sequences of siRNAs

| Gene | Sense (5'-3') | Antisense (5'-3') |
|---|---|---|
| *Clk4*-rat-1023 | GCAUAGGCUGCAUUCUUAUTT | AUAAGAAUGCAGCCUAUGCTT |
| *Clk4*-rat-1351 | GCACCCUUUCUUUGACUUATT | UAAGUCAAAGAAAGGGUGCTT |
| *Clk1*-rat-655 | GGUGGAGUGUAUCGAUCAUTT | AUGAUCGAUACACUCCACCTT |
| *Clk2*-rat-978 | CCACAUGUGUAUCUCCUUUTT | AAAGGAGAUACACAUGUGGTT |
| *Clk3*-rat-641 | GGAAGCUGCUCGUCUAGAATT | UUCUAGACGAGCAGCUUCCTT |
| *Pdha1*-rat-633 | GCUAACCAGGGUCAGAUAUTT | AUAUCUGACCCUGGUUAGCTT |
| *Nexn*-rat-790 | GCAAGGCGGAACAUGGUAATT | UUACCAUGUUCCGCCUUGCTT |
| *Nexn*-rat-246 | GGAAGAAGAGCCGUCUAAATT | UUUAGACGGCUCUUCUUCCTT |
| *Snw1*-rat-813 | CCAACUGGAAGAACGCUAATT | UUAGCGUUCUUCCAGUUGGTT |
| *Dusp27*-rat-2518 | GCCUCUGAUAACAAGCGUATT | UACGCUUGUUAUCAGAGGCTT |
| *Zc3hc1*-rat-733 | GCAAAUUAGGCUCAGACAUTT | AUGUCUGAGCCUAAUUUGCTT |
| *Ndrg1*-rat-514 | CCUUCACAAGUUUGGGCUUTT | AAGCCCAAACUUGUGAAGGTT |
| *CLK4*-homo-562 | GGAGACGUUCUAAGAGCAATT | UUGCUCUUAGAACGUCUCCTT |
| *CLK4*-homo-1400 | GCAAACCGUUGAAGGAAUUTT | AAUUCCUUCAACGGUUUGCTT |

**Supplementary Table 2** The primer pairs used for quantitative real-time PCR analysis of gene expression.

| Gene | Forward (5'-3') | Reverse (5'-3') |
|---|---|---|
| Mus-*Gapdh* | TGAAGGTCGGTGTGAACGGATTTGG | ACGACATACTCAGCACCAGCATCAC |
| Mus-*Clk4* | TGATCACATCAGGCAAATGGC | TCAATGTGCGCTCATCTCGT |
| Mus-*Nexn* | AGCCCCAAACCACATAACCA | GCCAAGCTTTGGCACATAGG |
| Mus-*Nppa* | GAACCTGCTAGACCACCT | CCTAGTCCACTCTGGGCT |
| Mus-*Nppb* | AAGCTGCTGGAGCTGATAAGA | GTTACAGCCCAAACGACTGAC |
| Mus-*Nyh7* | GCATCAAGGAGCTCACC | CTGCAGCCGCAGTAGGTT |
| Rat-*Clk4* | ACAGGGCTCCAGAGGTCATT | AGTATCCGCTCCATCATTGCC |
| Rat-*Gapdh* | AACGGCACAGTCAAGGCTGA | ACGCCAGTAGACTCCACGACAT |
| Rat-*Nppa* | CAACACAGATCTGATGGATTTCA | CCTCATCTTCTACCGGCATC |
| Rat-*Nppb* | GTCTCAAGACAGCGCCTTCC | AACCTCAGCCCGTCACAGC |
| Rat-*Myh7* | ATCAAGGGAAAGCAGGAAGC | CCTTGTCTACAGGTGCATCA |
| Homo-*CLK4* | ATCGTGGACACTTTGGGTGA | TCTAGCATCTGGACACATCGG |
| Homo-*NPPA* | ACAATGCCGTGTCCAACGCAGA | CTTCATTCGGCTCACTGAGCAC |
| Homo-*NPPB* | TCTGGCTGCTTTGGGAGGAAGA | CCTTGTGGAATCAGAAGCAGGTG |
| Homo-*GAPDH* | AGCCACATCGCTCAGACAC | GCCCAATACGACCAAATCC |

**Supplementary Table 3** Primers used for genotyping

| Gene target | Forward (5'-3') | Reverse (5'-3') |
|---|---|---|
| *Upstream loxP site** | AGGACACTCATCCCAGGTAGA | CCAACTGACCTTGGGCAAGAACAT |
| *Downstream loxP site** | TCTGAGGCGGAAAGAACCAG | ACACCCAAGTCAAGGACCTTAC |
| *3' loxP site*# | AATGTGGGACAGTCTAGGCAT | ATAGTCAACAGGGAGGACTTCC |
| *Cre (transgene)* | CTCCTCTTCCTGCCTGTT | TCTTGCGAACCTCATCAC |

*Primers used for sequencing to confirm precise insertion of loxP sites; #Primers used for simple genotyping.

**Supplementary Table 4** statistics of newborn mice*

| genotype | actual number | estimated number | male/female |
|---|---|---|---|
| *Clk4*$^{wt/wt}$; *MCM*$^{-/-}$ | 49 (14.0%) | 44 (12.5%) | 26/23 |
| *Clk4*$^{fl/wt}$; *MCM*$^{-/-}$ | 85 (24.3%) | 88 (25.0%) | 46/39 |
| *Clk4*$^{fl/fl}$; *MCM*$^{-/-}$ | 36 (10.3%) | 44 (12.5%) | 16/20 |
| *Clk4*$^{wt/wt}$; *MCM*$^{+/-}$ | 40 (11.4%) | 44 (12.5%) | 20/20 |
| *Clk4*$^{fl/wt}$; *MCM*$^{+/-}$ | 93 (26.6%) | 88 (25.0%) | 43/50 |
| *Clk4*$^{fl/fl}$; *MCM*$^{+/-}$ | 47 (13.4%) | 44 (12.5%) | 24/23 |

*All mice are offspring from *Clk4*$^{fl/wt}$; MCM$^{+/-}$ × *Clk4*$^{fl/wt}$; MCM$^{-/-}$

**Supplementary Table 5** Echocardiographic analysis of mice at week 0, 1, 2, and 3 post tamoxifen initiation

| | **week 0** | | **week 1** | | **week 2** | | **week 3** | |
|---|---|---|---|---|---|---|---|---|
| | **MCM (n=7)** | ***Clk4*-cKO (n=8)** | **MCM (n=7)** | ***Clk4*-cKO (n=8)** | **MCM (n=7)** | ***Clk4*-cKO (n=8)** | **MCM (n=7)** | ***Clk4*-cKO (n=8)** |
| **LVID, d (mm)** | 3.66 ± 0.02 | 3.71 ± 0.02 | 3.64 ± 0.02 | 3.67 ± 0.02 | 3.72 ± 0.02 | 3.73 ± 0.04 | 3.64 ± 0.03 | 3.90 ± 0.03[a] |
| **LVID, s (mm)** | 2.71 ± 0.04 | 2.86 ± 0.05 | 2.68 ± 0.05 | 2.77 ± 0.03 | 2.80 ± 0.05 | 2.70 ± 0.04 | 2.69 ± 0.07 | 3.17 ± 0.05 |
| **LVPW, d (mm)** | 0.61 ± 0.01 | 0.51 ± 0.02 | 0.67 ± 0.01 | 0.75 ± 0.01 | 0.63 ± 0.01 | 0.68 ± 0.02 | 0.64 ± 0.02 | 0.59 ± 0.01 |
| **LVPW, s (mm)** | 0.96 ± 0.04 | 0.89 ± 0.03 | 1.05 ± 0.03 | 1.11 ± 0.02 | 1.04 ± 0.03 | 1.24 ± 0.03 | 0.97 ± 0.04 | 0.95 ± 0.03 |
| **EF (%)** | 52.31 ± 1.11 | 46.17 ± 0.57 | 52.24 ± 0.90 | 49.69 ± 0.75 | 49.50 ± 0.49 | 56.17 ± 1.18 | 51.32 ± 1.23 | 37.82 ± 0.92[b] |
| **FS (%)** | 25.94 ± 0.61 | 22.83 ± 0.31 | 26.25 ± 0.51 | 24.55 ± 0.46 | 24.70 ± 0.21 | 27.72 ± 0.58 | 26.18 ± 0.61 | 18.65 ± 0.51 |
| **LV Mass (mg)** | 69.44 ± 2.47 | 60.70 ± 3.61 | 78.14 ± 2.35 | 91.20 ± 3.64 | 75.63 ± 2.12 | 83.98 ± 1.98 | 74.41 ± 2.25 | 76.37 ± 3.20 |
| **Heart Rate** | 473.03 ± 5.39 | 461.71 ± 7.01 | 470.23 ± 7.11 | 468.63 ± 7.24 | 472.88 ± 6.74 | 462.93 ± 6.11 | 449.56 ± 6.61 | 444.06 ± 5.86 |

Data are presented as the means ± S.E.M..[a]$P = 0.0463$, [b]$P = 0.0081$ vs MCM littermates (unpaired, two-tailed Student's t test.).

**Supplementary Table 6** Echocardiographic examination of *Clk4*-cKO mice treated with AAV-GFP/-WT/-S437E/-S437A

| | GFP | WT | S437E | S437A |
|---|---|---|---|---|
| **LVID, d (mm)** | 3.95 ± 0.02 | 3.93 ± 0.02 | 3.63 ± 0.02 | 4.13 ± 0.02 |
| **LVID, s (mm)** | 3.12 ± 0.05 | 3.14 ± 0.06 | 2.70 ± 0.04 | 3.33 ± 0.53 |
| **LVPW, d (mm)** | 0.68 ± 0.02 | 0.62 ± 0.02 | 0.72 ± 0.01 | 0.57 ± 0.03 |
| **LVPW, s (mm)** | 0.84 ± 0.05 | 0.79 ± 0.04 | 1.12 ± 0.04 | 0.81 ± 0.03 |
| **EF (%)** | 42.73 ± 1.49 | 38.79 ± 1.18 | 54.66 ± 0.88 | 39.12 ± 1.56 |
| **FS (%)** | 21.33 ± 0.67 | 20.13 ± 0.41 | 26.98 ± 0.54 | 19.42 ± 0.56 |
| **LV Mass (mg)** | 91.50 ± 3.10 | 81.40 ± 4.61 | 86.62 ± 2.78 | 79.71 ± 4.44 |
| **Heart Rate** | 481.88 ± 2.03 | 477.77 ± 2.11 | 479.71 ± 2.18 | 482.46 ± 2.71 |

Data are means ± S.E.M.

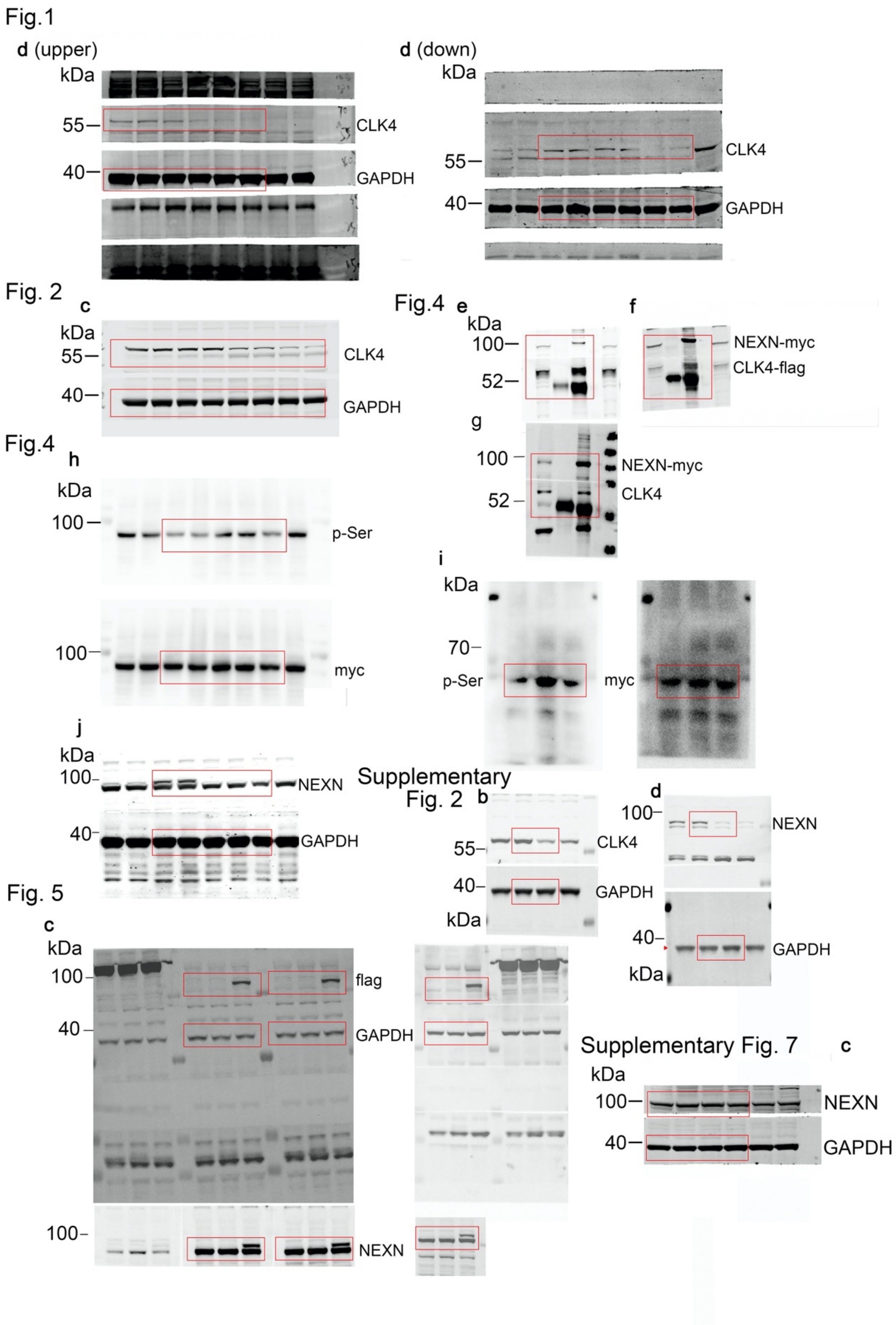


**Supplementary Fig. 11** Uncropped images from Western blots.
