# Supplementary material for: CDC-like kinase 4 deficiency contributes to pathological cardiac hypertrophy by modulating NEXN phosphorylation
Source: Nat Commun. 2022 Jul 30;13:4433. doi: 10.1038/s41467-022-31996-9 (PMC9338968; doi:10.1038/s41467-022-31996-9)
Supplement: Supplementary file 2 — Reporting Summary [file 41467_2022_31996_MOESM2_ESM.pdf]

nature portfolio

Corresponding author(s): Yi-Han Chen
Last updated by author(s): Jun 9, 2022

Reporting Summary

Nature Portfolio wishes to improve the reproducibility of the work that we publish. This form provides structure for consistency and transparency in reporting. For further information on Nature Portfolio policies, see our Editorial Policies and the Editorial Policy Checklist.

Statistics

For all statistical analyses, confirm that the following items are present in the figure legend, table legend, main text, or Methods section.

n/a	Confirmed	
☐	☒	The exact sample size (*n*) for each experimental group/condition, given as a discrete number and unit of measurement
☐	☒	A statement on whether measurements were taken from distinct samples or whether the same sample was measured repeatedly
☐	☒	The statistical test(s) used AND whether they are one- or two-sided<br>*Only common tests should be described solely by name; describe more complex techniques in the Methods section.*
☒	☐	A description of all covariates tested
☒	☐	A description of any assumptions or corrections, such as tests of normality and adjustment for multiple comparisons
☐	☒	A full description of the statistical parameters including central tendency (e.g. means) or other basic estimates (e.g. regression coefficient) AND variation (e.g. standard deviation) or associated estimates of uncertainty (e.g. confidence intervals)
☐	☒	For null hypothesis testing, the test statistic (e.g. *F*, *t*, *r*) with confidence intervals, effect sizes, degrees of freedom and *P* value noted<br>*Give P values as exact values whenever suitable.*
☒	☐	For Bayesian analysis, information on the choice of priors and Markov chain Monte Carlo settings
☒	☐	For hierarchical and complex designs, identification of the appropriate level for tests and full reporting of outcomes
☒	☐	Estimates of effect sizes (e.g. Cohen's *d*, Pearson's *r*), indicating how they were calculated

Our web collection on statistics for biologists contains articles on many of the points above.

Software and code

Policy information about availability of computer code

Data collection	No custom computer code or algorithm were used in the data analysis of this manuscript. Echocardiography: Vevo 2100 Imaging System. qPCR: QuantStudio Real-Time PCR System (Applied Biosystems). Microscopy: Leica DMI3000 B fluorescence microscope, Leica Application Suite v3.8. Phosphoproteomic profiling: Thermo Scientific EASY-nLC 1200 system.
Data analysis	No custom computer code or algorithm were used in the data analysis of this manuscript. Quantification of immunoblots and IICC/IF: Image J (Version 1.51m9). Statistical analyses for echocardiographic data, histological quantification and qPCR : GrapPad Prism 8. Phosphoproteomic analysis: Proteome Discoverer v2.2 (PD2.2, Thermo) ; Microsoft Excel (version16.61.1); KEGG Automatic Annotation Server (online version);

For manuscripts utilizing custom algorithms or software that are central to the research but not yet described in published literature, software must be made available to editors and reviewers. We strongly encourage code deposition in a community repository (e.g. GitHub). See the Nature Portfolio guidelines for submitting code & software for further information.

Data

Policy information about availability of data

All manuscripts must include a data availability statement. This statement should provide the following information, where applicable:

- Accession codes, unique identifiers, or web links for publicly available datasets
- A description of any restrictions on data availability
- For clinical datasets or third party data, please ensure that the statement adheres to our policy

The raw MS data generated in this study have been deposited to the ProteomeXchange Consortium via the PRIDE partner repository under accession code PXD029682 [https://www.ebi.ac.uk/pride/archive/projects/PXD029682]. The reference number for the mouse Swiss-Prot database used in this study is UP000000589. Microarray data have been deposited to the GEO database with the GEO accession number GSE188360 [https://www.ncbi.nlm.nih.gov/geo/query/

acc.cgi?acc=GSE188360]. Analyzed data generated in this study are provided in the Supplementary Information and Source Data file. Full scans of the blots are shown in Supplementary Fig. 11. Source data are provided with this paper.

Field-specific reporting

Please select the one below that is the best fit for your research. If you are not sure, read the appropriate sections before making your selection.

- [x] Life sciences
- [ ] Behavioural & social sciences
- [ ] Ecological, evolutionary & environmental sciences

For a reference copy of the document with all sections, see nature.com/documents/nr-reporting-summary-flat.pdf

Life sciences study design

All studies must disclose on these points even when the disclosure is negative.

Sample size	Required sample sizes were determined based on previous publications (Eric M et al Science 2016, Dong I et al Nature 2017, Mark J et al Nature 2019, Davy et al Nature communications 2021) and our past experience.
Data exclusions	No data were excluded from the analyses.
Replication	Replicates were used in in vivo experiments as noted in figure legends. In vitro experiments were performed at least three times independently. All attempts at replication were successful.
Randomization	All animals and cells were randomly assigned to the experimental group.
Blinding	Whenever possible, data were analyzed blind to condition. Data that require an automated analysis pipeline were not analyzed blind to conditions, including proteomic data and microarray data.

Reporting for specific materials, systems and methods

We require information from authors about some types of materials, experimental systems and methods used in many studies. Here, indicate whether each material, system or method listed is relevant to your study. If you are not sure if a list item applies to your research, read the appropriate section before selecting a response.

Materials & experimental systems

n/a	Involved in the study	
[ ]	[x]	Antibodies
[ ]	[x]	Eukaryotic cell lines
[x]	[ ]	Palaeontology and archaeology
[ ]	[x]	Animals and other organisms
[x]	[ ]	Human research participants
[x]	[ ]	Clinical data
[x]	[ ]	Dual use research of concern

Methods

n/a	Involved in the study	
[x]	[ ]	ChIP-seq
[x]	[ ]	Flow cytometry
[x]	[ ]	MRI-based neuroimaging

Antibodies

Antibodies used	anti-CLK4 rabbit polyclonal antibody (Abcam, ab104321, 1: 500), anti-GAPDH mouse monoclonal antibody (Proteintech, 60004-1-Ig, 1: 8000), anti-NEXN rabbit polyclonal antibody (Abcam, ab233235, 1: 500), anti-flag rabbit polyclonal antibody (Sigma, F7425, 1: 5000), anti-myc mouse monoclonal antibody (Cell Signaling Technology, 2276, 1: 1000) , Anti-Phosphoserine Antibody mouse monoclonal antibody (Sigma, 05-1000X, 1:1000), Mouse monoclonal [1C11] to Cardiac Troponin T (Abcam, ab8295, 1:200), Alexa Fluor 680-conjugated anti-mouse (Invitrogen, A32729, 1: 10000), Alexa Fluor 800-conjugated anti-rabbit (Invitrogen, A32735, 1: 10000)
Validation	All following primary antibodies used in this study were validated and were cited in the litterature.<br>anti-CLK4: Ajiro M et al. Therapeutic manipulation of IKBKAP mis-splicing with a small molecule to cure familial dysautonomia. Nat Commun 12, 4507 (2021).<br>anti-GAPDH: Liu et al. Sox17 is required for endothelial regeneration following inflammation-induced vascular injury. Nat Commun. 10,2126 (2019).<br>anti-NEXN: Hu Y et al. Long noncoding RNA NEXN-AS1 mitigates atherosclerosis by regulating the actin-binding protein NEXN. J Clin Invest. 129, 1115-1128 (2019).<br>anti-flag: Zekonyte U et al. Mitochondrial targeted meganuclease as a platform to eliminate mutant mtDNA in vivo. Nat Commun 12, 3210 (2021).<br>anti-myc: Gallardo-Montejano V et al. Perilipin 5 links mitochondrial uncoupled respiration in brown fat to healthy white fat remodeling and systemic glucose tolerance. Nat Commun 12, 3320 (2021).<br>anti-Phosphoserine : Zhang M et al. CaMKII-δ9 promotes cardiomyopathy through disrupting UBE2T-dependent DNA repair. Nat Cell

Biol 21, 1152–1163 (2019).
anti-Cardiac Troponin T: Liang D et al. Cellular and molecular landscape of mammalian sinoatrial node revealed by single-cell RNA sequencing. Nat Commun 12:287 (2021).

Eukaryotic cell lines

Policy information about cell lines

Cell line source(s)	hiPSC-derived CMs were purchased from Help Stem Cell Innovations (Nanjing, China).<br>The HEK293 cell lines were from Key Laboratory of Arrhythmias of the Ministry of Education of China, Tongji University School of Medicine, Shanghai, China.
Authentication	hiPSC-derived CMs were authenticated prior to the experiments with flow cytometry, immunofluorescence staining and patch clamp.
Mycoplasma contamination	We have confirmed that the cells were tested negative for mycoplasma contamination.
Commonly misidentified lines<br>(See ICLAC register)	No commonly misidentified cell lines were used in this study.

Animals and other organisms

Policy information about studies involving animals; ARRIVE guidelines recommended for reporting animal research

Laboratory animals	All mice were in the C57BL/6J background. Male 6-8 weeks old α-MHC-MerCreMer Clk4 wt/wt, α-MHC-MerCreMer Clk4 fl/fl, Clk4 fl/fl and wild-type mice were used. Cardiac-specific Clk4 knockout (Clk4-cKO) mice were generated by tamoxifen administration in α-MHC-MerCreMer Clk4 fl/fl mice. Neonatal Sprague Dawley rats were used for neonatal rat ventricular myocytes isolation.
Wild animals	Not involved.
Field-collected samples	Not involved.
Ethics oversight	The mice were fed ad libitum and housed at constant ambient temperature in a 12-h light cycle. All animal care and experimental protocols were performed in accordance with the 8th edition of the Guide for the Care and Use of Laboratory Animals and were approved by the Institutional Animal Care and Use Committee of the Tongji University School of Medicine (Shanghai, China).

Note that full information on the approval of the study protocol must also be provided in the manuscript.
