# Supplementary material for: CDC-like kinase 4 deficiency contributes to pathological cardiac hypertrophy by modulating NEXN phosphorylation
Source: Nat Commun. 2022 Jul 30;13:4433. doi: 10.1038/s41467-022-31996-9 (PMC9338968; doi:10.1038/s41467-022-31996-9)
Supplement: Supplementary file 3 — Description of Additional Supplementary Files [file 41467_2022_31996_MOESM3_ESM.pdf]

Description of Additional Supplementary files

File Name: Supplementary Data 1
Description: Data lists of quantitative phosphoproteomic analysis. The full description and datasets for the phosphoproteomic experiments were listed and statistical analysis and n numbers for all comparisons were shown.

File Name: Supplementary Data 2
Description: Data lists of Microarray analysis. The full description and datasets for the Microarray experiments were listed and statistical analysis and n numbers for all comparisons were shown.
